# Substrate-dependent incorporation of 15-lipoxygenase products in glycerophospholipids: 15-HETE and 15-HEPE in PI, 17-HDHA in plasmalogen PE, and 13-HODE in PC

**DOI:** 10.1016/j.jlr.2025.100841

**Published:** 2025-06-14

**Authors:** Laura Carpanedo, Luca M. Wende, Bjarne Goebel, Ann-Kathrin Häfner, Michel André Chromik, Nadja Kampschulte, Dieter Steinhilber, Nils Helge Schebb

**Affiliations:** 1 Chair of Food Chemistry, Faculty of Mathematics and Natural Sciences, University of Wuppertal, Wuppertal, Germany; 2 Institute of Pharmaceutical Chemistry, Goethe University Frankfurt, Frankfurt, Germany

**Keywords:** oxidized lipids, lipidomics, cell signaling, inflammation, arachidonic acid, oxylipin incorporation, mass spectrometry

## Abstract

Several oxylipins including hydroxy-PUFAs act as lipid mediators. In biological samples, the major part occurs esterified in glycero-phospholipids (PLs) or other lipids. In this work, the incorporation into glycero-PLs of 15-hydroxyeicosatetraenoic acid (15(*S*)-HETE), 15(*S*)-hydroxyeicosapentaenoic acid (15(*S*)-HEPE), 17(*S*)-hydroxydocosahexaenoic acid (17(*S*)-HDHA), and 13-(*S*)-hydroxyoctadecadienoic acid (13(*S*)-HODE) was investigated in oxylipin-supplemented human embryonic kidney 293T cells and cells overexpressing 15-lipoxgenase-2 (15-LOX-2, *ALOX15B*). Indirect quantification of esterified oxylipins in lipid fractions showed that >97% of each supplemented 15-LOX-2 product is esterified and that <25% are bound to neutral lipids, whereas >75% are bound to distinct glycero-PL classes, depending on the hydroxy-PUFA. 15-HETE and 15-HEPE were found in phosphatidylinositol (PI)/phosphatidylserine, whereas 17-HDHA was in phosphatidylethanolamine (PE) and 13-HODE in phosphatidylcholine (PC). The same pattern was found for oxylipins endogenously formed by overexpression of 15-LOX-2. A new targeted method for the analysis of oxidized glycero-PLs enabled to pinpoint the specific molecular species of the oxylipins. 15-HETE (20:4;15OH) and 15-HEPE (20:5;15OH) are dominantly found as PI 18:0/20:4;15OH (70%) and PI 18:0/20:5;15OH (80%), respectively. This preferential incorporation of 20:4;15OH and 20:5;15OH into PI may be biologically relevant for PI signaling pathways. In contrast, >50% of 17-HDHA (22:6;17OH) was found in PE P-16:0/22:6;17OH, PE P-18:0/22:6;17OH, and PE P-18:1/22:6;17OH. At least 40% of 13-HODE (18:2;13OH) was incorporated into PC 16:0/18:2;13OH, and relevant amounts were found in PI 18:0/18;13OH, PC 18:1/18;13OH, and PC-O (ether PC) 16:0/18;13OH. These results indicate that hydroxy-PUFAs are bound to glycero-PLs in a specific manner. The distinct incorporation of 15-LOX-2 products from different PUFAs into glycero-PLs might contribute to the biological effect of these oxylipins and their precursor FAs.

Eicosanoids and other oxylipins are formed by oxidation of PUFA (1, 2). Hydroxy-PUFAs can be obtained by the reduction of hydroperoxy-PUFA, which are generated nonenzymatically via reactive oxygen species, or enzymatically by cyclooxygenase, cytochrome P450 monooxygenases, and lipoxygenase (LOX) enzymes, such as 15-LOX-1, 15-LOX-2, 5-LOX, and 12-LOX (2, 3, 4). 15-LOX can oxidize both, nonesterified PUFAs as well as PUFAs esterified to glycero-phospholipids (PLs) (3, 5). Arachidonic acid (ARA) oxidation by 15-LOX-1 leads to the two products 15-hydro(pero)xyeicosatetraenoic acid (15(*S*)-H(p)ETE) and 12(*S*)-H(p)ETE, with pronounced species differences (6, 7). 15-LOX-2 converts ARA only to 15(*S*)-H(p)ETE (8). 15(*S*)-hydroxyeicosapentaenoic acid (HEPE), 17(*S*)-hydroxydocosahexaenoic acid (HDHA), and 13-(*S*)-hydroxyoctadecadienoic acid (13(*S*)-HODE) are generated by 15-LOX-2 from EPA, DHA, and linoleic acid, respectively (7). Moreover, both 15-HETE and 13-HODE can be formed in minor amounts by cyclooxygenase-2 (9, 10, 11) and cytochrome P450 monooxygenase enzymes (12). 15(*S*)-HETE and in part 13(*S*)-HODE are discussed to act predominantly anti-inflammatory (1, 13) and were found for example to have antitumor roles in smokers with non-small cell lung carcinoma (14). However, increased levels were also observed in inflammation, and higher levels of 15-HETE and 13-HODE were found in atherosclerotic plaques (15, 16). 15-HEPE may exhibit inflammatory effects and was found to protect wild-type mice against chemically induced colitis (17). Also, 17(*S*)-HDHA prevented hyperhomocysteinemia-induced formation of the nucleotide-binding oligomerization domain-like receptor containing pyrin domain 3 inflammasome in podocytes of mice and inhibited the formation of the proinflammatory cytokine interleukin-1β (18).

The major part of hydroxy-PUFA occurs in biological samples esterified in lipids such as PL or neutral lipids (NLs) (13, 19, 20, 21, 22). In the last decades, the quantification of esterified oxylipins was carried out indirectly by quantitative analysis of nonesterified oxylipins using LC coupled to targeted MS/MS following the cleavage of the ester bond by alkaline hydrolysis (23, 24). This analysis does not provide the information in which lipid classes/species and at which *sn*-position oxylipins are located. Thus, it is not possible to study the biological roles of oxidized PLs (oxPLs), including PLs bearing 15-HETE (20:4;15OH), 15-HEPE (20:5;15OH), 17-HDHA (22:6;17OH), and 13-HODE (18:2;13OH).

In the 1990s, incorporation of 15-HETE and 13-HODE into lipids was investigated using TLC following supplementation of cells with radioactively labeled oxylipins, for example, [^3^H]15-HETE or [^14^C]15-HETE (25, 26, 27, 28, 29, 30, 31, 32). This approach provided a first insight in which lipid classes these oxylipins are incorporated following supplementation but could not provide information in which molecular species they are esterified. Moreover nonlabeled oxylipins and thus endogenous formation could not be investigated. In the 2010s, the main LOX products from ARA, i.e., 15-HETE, 12 -HETE, and 5-HETE, have been detected in different PL species by LC-MS in human monocytes (33), in murine peritoneal macrophages from naïve lavage (34), human platelets (33, 35), and human neutrophils (36) by O’Donnell and coworkers.

In this study, we aimed to characterize in which PL classes dominating 15-LOX products from the main PUFAs are esterified. For this purpose, we investigated the pattern of oxylipins in human embryonic kidney 293T (HEK293T) cells following either exogenous oxylipin supplementation or endogenous oxylipin formation by 15-LOX-2 using a genetically modified cell line. The vast majority of oxylipins was found esterified in HEK293T cells, predominantly in PL. Hydroxy-PUFAs exhibited distinct incorporation patterns across PL classes. Using a newly developed targeted LC-MS/MS method, each hydroxy-PUFA was found to be specifically esterified into distinct molecular PL species. Supplemented and 15-LOX-2-overexpressing HEK293T cells showed a similar incorporation pattern of 15-LOX products into PL classes and species. The dramatic differences between the incorporation pattern of the 15-LOX products of different PUFAs might be relevant for phosphatidylinositol (PI) signaling pathways.

## Materials and methods

### Chemicals and biological material

PI 12:0/13:0, phosphatidylglycerol (PG) 12:0/13:0, phosphatidylcholine (PC) 12:0/13:0, and phosphatidylethanolamine (PE) 12:0/13:0 were purchased from Avanti Polar Lipids (local supplier: Merck, Darmstadt, Germany). The standards PC 16:0/20:4(5*Z*,8*Z*,11*Z*,14*Z*), PC 18:0/20:4 (5*Z*,8*Z*,11*Z*,14*Z*), PE 18:0/20:4(5*Z*,8*Z*,11*Z*,14*Z*), PC 18:0/20:5(5*Z*,8*Z*,11*Z*,14*Z*,17*Z*), PC 16:0/22:6(4*Z*,7*Z*,10*Z*,13*Z*,16*Z*,19*Z*), PC 18:0/22:6(4*Z*,7*Z*,10*Z*,13*Z*,16*Z*,19*Z*), PE 16:0/22:6(4*Z*,7*Z*,10*Z*,13*Z*,16*Z*,19*Z*), PC 18:0/22:4(7*Z*,10*Z*,13*Z*,16*Z*), and PE 18:0/22:4(7*Z*,10*Z*,13*Z*,16*Z*) as well as 15(*S*)-HETE, 15(*S*)-HEPE, 17(*S*)-HDHA, and 13(*S*)-HODE were from Cayman Chemical (Ann Arbor, MI; local supplier: Biomol, Hamburg, Germany). Ultrapure water (18.2 MΩ cm) was generated using the Barnstead GenPure Pro system from Thermo Fisher Scientific (Langenselbold, Germany). Ammonium formate was supplied by Sigma-Aldrich (Schnelldorf, Germany). Soybean 15-LOX (type IB) and all other chemicals were purchased from Merck.

HEK293T cells were obtained from the German Collection of Microorganisms and Cell Cultures GmbH (DSMZ, Braunschweig, Germany). Transfected HEK293T cells with doxycycline-inducible 15-LOX-2 expression using a sleeping beauty system characterized in Ref. (19) were used.

### Preparation of oxidized glycero-PL standards by soybean 15-LOX-1

OxPL standards were generated based on a protocol from Morgan *et al*. (37). Briefly, individual PL standards were dried using nitrogen, then resuspended in 40 mM borate buffer (pH = 9.0) and 10 mM deoxycholate to a final concentration of 0.1 mM. Soybean 15-LOX was added (5 kU/ml), and samples were incubated at room temperature for 2 h. Hydroperoxy-PUFAs were reduced to the corresponding hydroxy-PUFAs by adding 1.3 μmol of tin(II) chloride in H_2_O, and lipids were extracted using a mixture of methanol and methyl tert-butyl ether. The oxidized product was separated from its unoxidized precursor using reversed-phase LC coupled to an ultraviolet spectroscopic detector. Chromatographic separation was achieved on a LiChrospher 100 C18 column (4.6 × 250 mm, 5 μm, 95 Å; Bischoff) with a flow rate of 1 ml/min. A binary gradient (68% B to 100% B over 15 min, hold 100% B for 20 min) was used with eluent A (H_2_O/methanol, 95:5, v/v) and eluent B (methanol/H_2_O, 99:1, v/v/v), both containing 10 mM ammonium formate. Elution of oxPL was monitored at 235 nm and the PL precursor at 205 nm. The concentration of the purified oxPL standards was determined by quantitative targeted LC-MS/MS analysis of oxylipins following alkaline hydrolysis (24, 38, 39, 40).

### Lipid extraction

A single cell pellet was used for the parallel analysis of total and nonesterified oxylipins, esterified oxylipins in lipid fractions, as well as intact oxPLs (Supplemental Fig. S1). Dry pellets were resuspended in 290 μl methanol/H_2_O (50:50, v/v), followed by the addition of 10 μl antioxidant/inhibitor mixture (10 μl 0.2 mg/ml butylated hydroxytoluene, 100 μM indomethacin, and 100 μM t-AUCB) (24, 38, 39, 40). Samples were vortexed and sonicated for 10 s. Protein content was determined from the cell homogenate by bicinchoninic acid assay (41, 42), and the concentrations of oxylipins and oxPLs were calculated based on the amount of cellular protein.

#### Solid-phase extraction

Nonesterified and total oxylipins were extracted using an established procedure (24, 38, 39, 40), using 50 μl and 100 μl of cell suspension, respectively (Supplemental Fig. S1A, B). Deuterium-labeled oxylipins (Cayman Chemicals; local supplier: Biomol, Hamburg, Germany) were added as internal standards (ISs). Protein precipitation was performed using 280 μl methanol for nonesterified oxylipins and 400 μl isopropanol for total oxylipins. After centrifugation (4 °C, 10 min, 20,000 *g*), the supernatant from the total oxylipin samples was hydrolyzed for 30 min at 60 °C with 100 μl 0.6 M KOH (H_2_O/methanol, 25:75, v/v) (Supplemental Fig. S1B) (24). Following alkaline hydrolysis, oxylipins were extracted using mixed-mode solid-phase extraction (SPE) with Bond Elut Certify II cartridges (C8 and anion exchange, 40 μm, 3 ml/200 mg; Agilent, Waldbronn, Germany) (24, 39). For the extraction of nonesterified oxylipins, the supernatant was directly applied to the SPE cartridges. After evaporation, the residues were reconstituted, sonicated, and analyzed by targeted metabolomics LC-MS/MS (24, 38, 39, 40). Additionally, lipids were fractionated into lipid classes using hydrophilic interaction liquid chromatography (HILIC)-based SPE cartridges (aminopropyl-modified silica; Macherey-Nagel, Düren, Germany) with 50 μl cell suspension (43, 44, 45) (Supplemental Fig. S1C). Following protein precipitation, the supernatant was applied to the HILIC-based cartridges, and the lipid fractions—PI/PS, PE, PC, PG, and NL—were separated and collected (46). After evaporation, esterified oxylipins in the lipid fractions were quantified as described above.

#### Liquid-liquid extraction

Lipids were extracted from the HEK293T cells using a modified liquid-liquid extraction based on the study by Matyash *et al*. (47) with slight modifications described (48) (Supplemental Fig. S1D). Briefly, 50 μl suspension of sonicated cells (i.e., 0.38 mg protein) was transferred to 3 ml glass tubes. Around 2.5 pmol of each ISs (i.e., PI 12:0/13:0, PC 12:0/13:0, and PE 12:0/13:0) were added yielding a final concentration of 50 nM in the lipid extract. Lipids were extracted using a mixture of methanol and methyl *tert*-butyl ether. After extraction and evaporation, the dried residue was reconstituted in 50 μl of isopropanol/acetonitrile (50:50, v/v) containing 50 nM PG 12:0/13:0 as IS2 used to calculate extraction recovery of IS. The extracts were sonicated and analyzed by the targeted oxPL LC-MS/MS method.

### LC-MS/MS analysis

#### Targeted analysis of oxylipins

Targeted LC-MS/MS analysis of oxylipins was carried out using a 1290 Infinity II (Agilent) LC system coupled to a QTRAP 5500 mass spectrometer (Sciex, Darmstadt, Germany) as described (24, 38, 39, 40). The injection volume was 5 μl. Chromatographic separation was carried out on a ZORBAX Eclipse Plus C18 column (2.1 × 150 mm, 1.8 μm, 95 Å; Agilent) equipped with a guard column (2.1 × 2 mm, 1.8 μm) at 40°C. A binary gradient was used with eluent A (0.1% acetic acid in H_2_O/eluent B, 95:5, v/v) and eluent B (acetonitrile/methanol/acetic acid, 800:150:1, v/v/v). Oxylipins were separated using the following gradient with a flow rate of 300 μl/min: 0–1.0 min 21% B; 1.0–1.5 min 21–26% B; 1.5–10 min 26–51% B; 10–19 min 51–66% B; 19–25.1 min 66–98% B; 25.1–27.6 min 98% B; 27.6–27.7 min 21% B; and 27.7–31.5 min 21% B. MS detection of oxylipins was performed in scheduled selected reaction monitoring mode following negative ESI. Oxylipins were quantified using external calibrations with IS.

#### Untargeted analysis of oxidized glycero-PL

Untargeted LC-high resolution (HR)MS analysis of oxPLs was carried out as described (48) on a Vanquish Horizon high performance LC system coupled to a hybrid quadrupole-orbitrap mass spectrometer (Q Exactive HF; Thermo Fisher Scientific, Dreieich, Germany). The injection volume was 5 μl. Chromatographic separation was carried out on an ACQUITY Premier CSH C18 column (2.1 × 100 mm, 1.7 μm, 130 Å; Waters, Eschborn, Germany) equipped with a guard column (2.1 × 5 mm, 1.7 μm) at 40 °C. A binary gradient was used with eluent A (H_2_O/acetonitrile, 60:40, v/v) and eluent B (isopropanol/acetonitrile, 80:20, v/v, +1% H_2_O), both containing 10 mM ammonium formate and 0.1% formic acid. Lipids were separated using the following gradient with a flow rate of 260 μl/min: 0–0.7 min 30% B; 0.7–0.8 min 30–57.5% B; 0.8–9 min 57.5% B; 9–22 min 57.5–68% B; 22–24 min 68–99% B; 24–28 min 99% B; and 28–30 min 30% B. Lipids were analyzed in full MS data-dependent (dd) MS^2^ TOP 15 mode (full MS/ddMS^2^) following negative ESI mode. The full MS scans were recorded over a mass range of *m/z* 200–1,200 at a resolution setting of 120,000, and ddMS^2^ spectra were acquired at a resolution setting of 15,000. For data acquisition and instrument control, Chromeleon software (version 7.2.11; Thermo Fisher Scientific) was used. OxPL species were characterized as described in Ref. (48) based on precursor ion, product ions, and retention time using Freestyle software (version 1.8; Thermo Fisher Scientific).

#### Targeted analysis of oxidized glycero-PL

Targeted LC-MS/MS analysis of oxPLs was carried out using a 1290 Infinity II (Agilent) LC system coupled to a QTRAP 6500+ mass spectrometer (Sciex). The injection volume was 5 μl. Lipids were separated using the ACQUITY Premier CSH C18 column with the same eluents and gradient conditions as the untargeted LC-HRMS method (see above). MS detection was performed in negative ESI mode with the following settings: ion spray voltage –4,500 V, source temperature 650°C, nebulizer gas (gas 1, compressed air purified with RAMS05Z; CMC instruments, Eschborn, Germany) 60 psi, and drying gas (gas 2, purified compressed air) 60 psi, curtain gas (nitrogen, generated with the nitrogen generator Eco Inert-ESP4; DWT, Bottrop, Germany) 35 psi, and collision gas (nitrogen) 6 psi. MS detection was carried out in scheduled multiple reaction monitoring mode acquiring three transitions per oxPL: two for qualification and one for quantification resulting from the specific cleavage of the oxidized fatty acyl chains. The detection window was set to 120 s around the retention time and the (maximum) cycle time to 1 s. The collision energy was optimized for each of the oxPL. The declustering potential, entrance potential, and collision cell exit potential were –50 V, –2 V, and –10 V, respectively, as these parameters have a minor impact on the signal intensity. MS parameters for targeted oxPL analysis can be found in Tables 1 and Supplemental Table S1. Analyst (Sciex, version 1.7) was used for instrument control and data acquisition, and Multiquant (Sciex, version 2.1.1) was used for data evaluation.

**Table 1:** Parameters of the targeted LC-ESI(−)-MS/MS method

Analyte	Mass transition		CE	tR
	Q1	Q3		(min)
PI 16:0/18:2;13OH	849.5	195.2	−65	8.19
PI 18:0/18:2;13OH	877.6	195.2	−65	11.95
PI 16:0/20:4;15OH	873.5	219.2	−55	8.63
PI 16:0/20:5;15OH	871.5	219.2	−55	7.41
PI 18:0/20:4;15OH	901.5	219.2	−55	12.62
PI 18:1/20:4;15OH	899.5	219.2	−55	8.95
PI 18:0/20:5;15OH	899.5	219.2	−55	10.77
PI 16:0/22:5;17OH	899.5	247.2	−55	9.01
PI 18:1/20:5;15OH	897.5	219.2	−55	7.67
PI 16:0/22:6;17OH	897.5	201.3	−55	8.34
PI 18:0/22:5;17OH	927.6	247.2	−60	13.07
PI 18:0/22:6;17OH	925.5	201.3	−60	12.19
PI 18:1/22:6;17OH	923.5	201.3	−55	8.64
PC 16:0/18:2;13OH	818.5	195.1	−60	10.80
PC 16:1/18:2;13OH	816.5	195.2	−60	7.75
PC 18:0/18:2;13OH	846.6	195.1	−60	15.30
PC 18:1/18:2;13OH	844.6	195.1	−60	11.16
PC 16:0/15-HETE	842.4	219.2	−50	11.42
PC 16:0/20:5;15OH	840.5	219.2	−50	9.64
PC 18:0/15-HETE	870.6	219.2	−55	15.99
PC 16:0/22:5;17OH	868.5	247.2	−50	11.93
PC 18:0/15-HEPE	868.5	219.2	−50	14.04
PC 18:1/20:5;15OH	866.4	219.2	−50	10.00
PC 16:0/17-HDHA	866.4	201.3	−50	11.02
PC 18:0/DH-17-HETE	898.5	247.3	−55	18.43
PC 18:1/22:5;17OH	894.5	247.2	−55	12.33
PC 18:0/17-HDHA	894.5	201.3	−55	15.54
PC 18:1/22:6;17OH	892.6	201.3	−50	11.39
PC P-16:0/18:2;13OH	802.6	195.1	−60	12.55
PC P-16:0/20:4;15OH	826.6	219.2	−50	13.16
PC P-16:0/22:5;17OH	852.6	247.2	−55	13.68
PC O-16:0/18:2;13OH	804.6	195.1	−60	13.27
PC O-16:0/20:4;15OH	828.6	219.2	−50	13.90
PC O-18:1/20:4;15OH	854.6	219.2	−55	14.22
PC O-16:0/22:5;17OH	854.6	247.2	−55	14.43
PC O-16:0/22:6;17OH	852.6	201.3	−50	13.44
PC O-18:1/22:5;17OH	880.6	247.2	−55	14.72
PC O-18:1/22:6;17OH	878.6	201.3	−60	13.72
PE 16:0/18:2;13OH	730.5	195.2	−50	11.76
PE 18:0/18:2;13OH	758.5	195.2	−55	16.33
PE 16:0/20:4;15OH	754.4	219.2	−45	12.39
PE 16:0/20:5;15OH	752.4	219.2	−40	10.48
PE 18:0/15-HETE	782.5	219.2	−45	17.01
PE 18:1/20:4;15OH	780.5	219.2	−45	12.84
PE 18:0/20:5;15OH	780.5	219.2	−45	15.03
PE 18:1/20:5;15OH	778.4	219.2	−45	10.91
PE 16:0/17-HDHA	778.4	201.3	−45	11.97
PE 18:0/DH-17-HETE	810.7	247.1	−48	19.49
PE 18:0/22:5;17OH	808.5	247.2	−45	17.50
PE 18:0/22:6;17OH	806.5	201.3	−45	16.56
PE 18:1/22:5;17OH	806.5	247.2	−50	13.35
PE 18:1/22:6;17OH	804.5	201.3	−45	12.40
PE P-16:0/18:2;13OH	714.5	195.2	−50	13.68
PE P-16:0/20:4;15OH	738.5	219.2	−45	14.28
PE P-16:0/20:5;15OH	736.5	219.2	−40	12.31
PE P-18:0/20:4;15OH	766.5	219.2	−45	18.96
PE P-16:0/22:4;17OH	766.5	247.2	−45	16.83
PE P-18:1/20:4;15OH	764.5	219.2	−45	14.63
PE P-18:0/20:5;15OH	764.5	219.2	−40	16.98
PE P-16:0/22:5;17OH	764.5	247.2	−45	14.85
PE P-18:1/20:5;15OH	762.5	219.2	−45	12.65
PE P-16:0/22:6;17OH	762.5	201.5	−45	13.80
PE P-18:0/22:4;17OH	794.6	247.2	−45	19.49
PE P-18:0/22:5;17OH	792.5	247.2	−45	19.49
PE P-18:1/22:5;17OH	790.5	247.2	−45	15.17
PE P-18:0/22:6;17OH	790.5	201.3	−45	18.43
PE P-18:1/22:6;17OH	788.5	201.3	−45	14.13
PI 12:0/13:0 (IS)	711.4	241.0	−48	7.36
PC 12:0/13:0 (IS)	680.4	213.0	−42	9.75
PE 12:0/13:0 (IS)	592.0	213.0	−38	10.73
PG 12:0/13:0 (IS)	623.2	213.0	−42	7.73

Shown are the mass transitions used for quantification in the scheduled multiple reaction monitoring method, collision energy (CE), and the retention time (tR). Transitions used for qualification are depicted in Supplemental Table S1.

For calibration, individual oxPL standards were mixed and diluted in acetonitrile/isopropanol (50:50, v/v) at 11 concentration levels (Supplemental Table S2). Each calibration level contained the same amount of the IS (PI 12:0/13:0, PC 12:0/13:0, and PE 12:0/13:0; 50 nM for each). Linear calibration was carried out using linear least squares regression (weighting: 1/x^2^). Analyte quantification was carried out based on the analyte to corresponding IS peak area ratio. All standards are listed in Supplemental Tables S2 and S3, and the concentration of oxPL in oxylipin-supplemented cells and in 15-LOX-2 overexpressing cells can be found in Supplemental Table S4.

### Method characterization of targeted analysis of oxidized glycero-PL

The LC-MS/MS method was characterized and validated regarding sensitivity (LOD and lower limit of quantification (LLOQ)), linearity, extraction recovery, intraday and interday accuracy and precision, and dilution integrity as described in the supplemental data (Supplemental Fig. S2–S6, and Supplemental Tables S2, S3, S5, S6, and S7) based on criteria of the Guideline on bioanalytical method validation of the International Council for Harmonization (49).

### Cell culture

HEK293T cells were cultivated in DMEM high glucose (4.5 g/l) supplemented with 10% (v/v) fetal calf serum, 100 U/ml penicillin, 100 μg/ml streptomycin, and 1 mM sodium pyruvate in a humidified atmosphere with 5% CO_2_ at 37 °C. In 10 cm^2^ dishes, 5 × 10^6^ cells were seeded and cultivated for 24 h to allow cell adhesion. Following 24 h, the medium was replaced with 10 ml of fresh serum-free medium. For supplementation, 10 μl of either 15(*S*)-HETE, 15(*S*)-HEPE, 17(*S*)-HDHA, or 13(*S*)-HODE in DMSO was directly spiked into the medium leading to a final concentration of 300 nM (0.1% DMSO) and incubated for 2 h (48). Cells were harvested by scraping in ice-cold PBS.

HEK293T cells were transfected with 15-LOX-2 using the sleeping beauty system (50) described in Ref. (19) using the pSBtetmChP_15LO2 plasmid for construct. This plasmid results in a silenced gene expression until doxycycline treatment and also contains the mCherry gene as a fluorescent marker and a puromycin resistance gene for positive selection. In a 6-well plate, 1 × 10^5^ HEK293T cells were seeded and let adhere for 24 h. The medium was replaced with 2 ml of fresh medium followed by the addition of the transfection mixture containing the plasmid (2.5 μg), a second plasmid carrying the SB100X transposase gene (0.25 μg), and polyethylenimine (12.5 μg). Stable transfection of cells was performed for 16 h. The medium was exchanged with fresh medium containing 2.5 µg/ml puromycin and homogenous mCherry expression was checked by fluorescence.

To induce 15-LOX-2 overexpression in transfected HEK293T_15-LOX-2 cells, 5 × 10^6^ cells were seeded and cultivated for 24 h to allow cell adhesion. The medium was replaced after 24 h with 10 ml of fresh medium containing 200 ng/ml doxycycline for 24 h (0.1% DMSO). Cells were harvested by scraping in ice-cold PBS. Overexpression of 15-LOX-2 in HEK293T_15-LOX-2 cells was characterized by quantification of the enzyme level of 15-LOX-2 and concentration levels of 15-LOX-2 oxylipin products using combined targeted LC-MS/MS proteomics (38, 51) and oxylipin metabolomics (24, 38, 39, 40) (Supplemental Fig. S7). The targeted proteomics LC-MS/MS method is described in the supplemental data section.

### Lipid notation

Oxylipins in human HEK293T cells were analyzed using a combination of indirect and direct LC-MS approaches (Supplemental Fig. S1). The terms “indirect quantification” or “indirect analysis” refer to the measurement of esterified oxylipins as nonesterified by targeted LC-MS/MS following alkaline hydrolysis (Supplemental Fig. S1B, C). The terms “direct quantification” or “direct analysis” refer to the measurement of esterified oxylipins as oxPL using untargeted LC-HRMS (48) or the targeted LC-MS/MS method developed in this work (Supplemental Fig. S1D).

The notations 15-HETE, 15-HEPE, 17-HDHA, and 13-HODE are used when identification was confirmed using authentic standards (i.e., oxylipin or oxPL), whereas the notations 20:4;15OH, 20:5;15OH, 22:6;17OH, and 18:2;13OH are used when authentic standards of the oxylipin-containing species were not available. Furthermore, the notations 15(*S*)-HETE, 15(*S*)-HEPE, 17(*S*)-HDHA, and 13(*S*)-HODE are used only to describe the supplemented (enantiopure) oxylipins.

## Results

### Incorporation of oxylipins into glycero-PL classes

Oxylipins—especially hydroxy-PUFAs—are mainly found esterified to lipids in biological samples (19, 20, 21, 52). However, information in which lipid class hydroxy-PUFAs are bound is scarce and has been described only in studies following oxylipin supplementation (25, 26, 27, 28, 29, 30, 31, 32, 48). We aimed to investigate in which PL classes of the HEK293T cell line the 15-LOX products 15(*S*)-HETE, 15(*S*)-HEPE, 17(*S*)-HDHA, and 13(*S*)-HODE are incorporated.

**Fig. 1:**
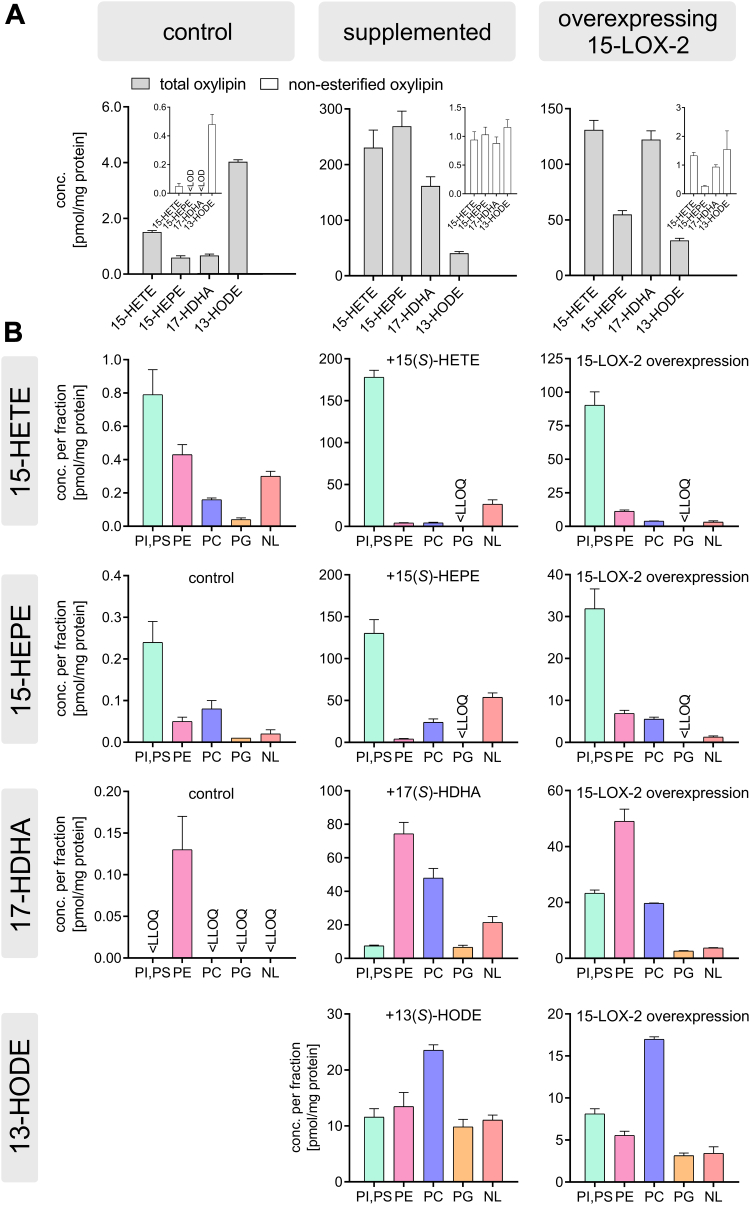
Incorporation of oxylipins in distinct lipid classes at baseline, following supplementation, and following overexpression of 15-LOX-2 in HEK293T cells. Around 5 × 10^6^ HEK293T cells were used per experiment. Left: HEK293T cells treated with 0.1% DMSO as controls. Middle: HEK293T cells supplemented with either 15(*S*)-HETE, 15(*S*)-HEPE, 17(*S*)-HDHA, or 13(*S*)-HODE (300 nM, 2 h). Right: Endogenous formation of oxPL elicited by overexpression of 15-LOX-2 in HEK293T_15-LOX-2 cells (200 ng/ml doxycycline, 24 h). Oxylipins were analyzed as nonesterified following alkaline hydrolysis by targeted LC-MS/MS method. Shown are the concentrations per milligram cellular protein of (A) nonesterified and total oxylipins and (B) esterified oxylipins in each HILIC-separated lipid fraction, (mean ± SD [n = 3]). At baseline concentration, 13-HODE is not shown because of interferences caused by a contamination occurring in the HILIC-based cartridges.

Quantitative targeted analysis unveiled that the baseline concentration of total 15-HETE, 15-HEPE, 17-HDHA, and 13-HODE is ≤4.0 pmol/mg protein, with nonesterified concentrations below 0.48 pmol/mg (Fig. 1A). Following supplementation (300 nM, 2 h), levels of the added hydroxy-PUFAs increased to 40–270 pmol/mg. Around 97% of 15-HETE and 15-HEPE occurred esterified and showed similar incorporation rates of 20 ± 2% and 21.4 ± 0.3% of the added amount, respectively. 17-HDHA and 13-HODE were also only detected esterified (>97%) but were incorporated with lower amounts (12.8 ± 0.7% and 3.4 ± 0.3%, respectively). Alpert *et al*. (29) also found a comparable incorporation rate of 29 ± 5% in human tracheal epithelial cells following a 2 h incubation with 1 μM of [^3^H]15(*S*)-HETE by TLC. Concentrations of hydroxy-PUFAs formed endogenously via 15-LOX-2 using a genetically modified HEK293T cell line were in the same order of magnitude. Most oxylipins (>97%) in the cells were esterified, which is consistent with our previous works showing that 15-HETE and other hydroxy-PUFAs such as 12-HETE and 5-HETE occur in cells esterified at baseline, following supplementation, and endogenous formation by 15-LOX (19, 48).

Using HILIC fractionation (43, 44, 45), we found that >75% of the hydroxy-PUFAs were bound to polar lipids, that is, lysoglycero-PLs (lyso-PLs) and PL, and >25% were esterified into NL, such as diacylgylcerols (DG), triacylglycerols, and cholesteryl esters (Fig. 1B). Following supplementation (300 nM, 2 h), incorporation of hydroxy-PUFAs in NL was higher compared with oxylipins endogenously formed by 15-LOX-2, especially for 15-HEPE with 25% in NL versus 2.8% in cells overexpressing 15-LOX-2. For 15-HETE, 17-HDHA, and 13-HODE, smaller differences (<10%) were found between supplementation and endogenous formation. This is consistent with earlier reports showing that 15-HETE incorporation in both PL and PI is saturable in human tracheal epithelial cells and that increased amounts of [H^3^]15-HETE were found in NL above 1 μM supplementation (29). In our study, the concentrations of 15-HETE and 15-HEPE following supplementation were higher compared with 15-LOX-2 endogenous formation, for example, for 15-HEPE with 270 pmol/mg versus 55 pmol/mg in 15-LOX-2-overexpressing cells (Fig. 1A). When a lower concentration was used for supplementation (i.e., ∼50 nM, 2 h), fewer hydroxy-PUFAs were incorporated in NL, and the proportion was similar to the one in 15-LOX-2-expressing cells (Supplemental Table S8). These results indicate the incorporation of 15-HETE and 15-HEPE into PL via specific mechanisms that can be saturated.

Oxylipins were distinctly incorporated into PL classes, that is, PI/PS, PE, and PC. Only very low amounts were bound to PG. Despite minor differences, the incorporation patterns of hydroxy-PUFAs into PL classes were comparable at baseline concentration, following supplementation, and in cells overexpressing 15-LOX-2 (Fig. 1B): The majority of 15-HETE and 15-HEPE were found in PI/PS, whereas more than half of 17-HDHA was detected in PE and half of 13-HODE was found in PC. Following supplementation, 178 ± 8 pmol/mg (95%) of 15-HETE was found in PI/PS and 90 ± 10 pmol/mg (84%) of endogenously formed 15-HETE was incorporated into PI/PS. Incorporation of 15-HETE into other PL classes was minor in both supplemented cells and 15-LOX-2-overexpressing cells, with 8.4 ± 0.9 pmol/mg versus 15 ± 1 pmol/mg, respectively, in the sum of PC and PE. 15-HEPE presented a similar distribution between the PL classes: 32 ± 5 pmol/mg (72%) of 15-HEPE was in PI/PS, 6.9 ± 0.7 pmol/mg in PE, and 5.5 ± 0.5 pmol/mg in PC in cells overexpressing 15-LOX-2. For both 15-HETE and 15-HEPE, incorporation into PE was higher in 15-LOX-2-overexpressing cells compared with cells supplemented with oxylipins: 11% and 16%, respectively, of endogenously formed 15-HETE and 15-HEPE was found in PE versus 2.0% and 1.6% following supplementation. At baseline concentration, 17-HDHA was only detected in PE likely because of its low concentration (0.13 pmol/mg), close to the LLOQ. Following supplementation and endogenous formation by 15-LOX-2, most 17-HDHAs were also found in PE, 74 ± 7 pmol/mg (55%) and 49 ± 4 pmol/mg (52%), respectively. One noticeable difference was observed in the incorporation pattern of 17-HDHA: endogenously formed 17-HDHA was more incorporated into PI/PS with 23 ± 1 pmol/mg (25%) versus 7.5 ± 0.4 pmol/mg (5.5%) following supplementation. PI/PS were also the second most abundant PL classes for 13-HODE incorporation with 8.1 ± 0.6 pmol/mg, but the major part of 13-HODE was detected in PC with 17.0 ± 0.3 pmol/mg (50%) in 15-LOX-2-overexpressing cells. In 13(*S*)-HODE-supplemented cells, 23 ± 1 pmol/mg (40%) and 12 ± 2 pmol/mg were found in PC and PI, respectively.

In this work, we demonstrate that 15-LOX products show a distinct incorporation pattern into PL, and we also show the incorporation of 17-HDHA and 15-HEPE into lipids, which was not previously described. Importantly, we demonstrate this pattern not only following supplementation, as carried out for labeled 15-HETE and 13-HODE in the 1990s (25, 26, 27, 28, 29, 30, 31, 32), but also for oxylipins endogenously formed by 15-LOX-2. Because our method also allows us to detect unlabeled lipids, we demonstrate that endogenously formed hydroxy-PUFAs, at baseline or upon 15-LOX-2 activity show a comparable incorporation pattern, highlighting the biological relevance of these findings.

### Direct analysis of oxidized glycero-PLs

Indirect analysis of oxPL in lipid fractions in HEK293T cells unveiled that 15-HETE, 15-HEPE, 17-HDHA, and 13-HODE are incorporated into specific polar lipid classes. Based on this finding, we aimed to characterize and quantify the PL molecular species into which the oxylipins are incorporated.

**Fig. 2:**
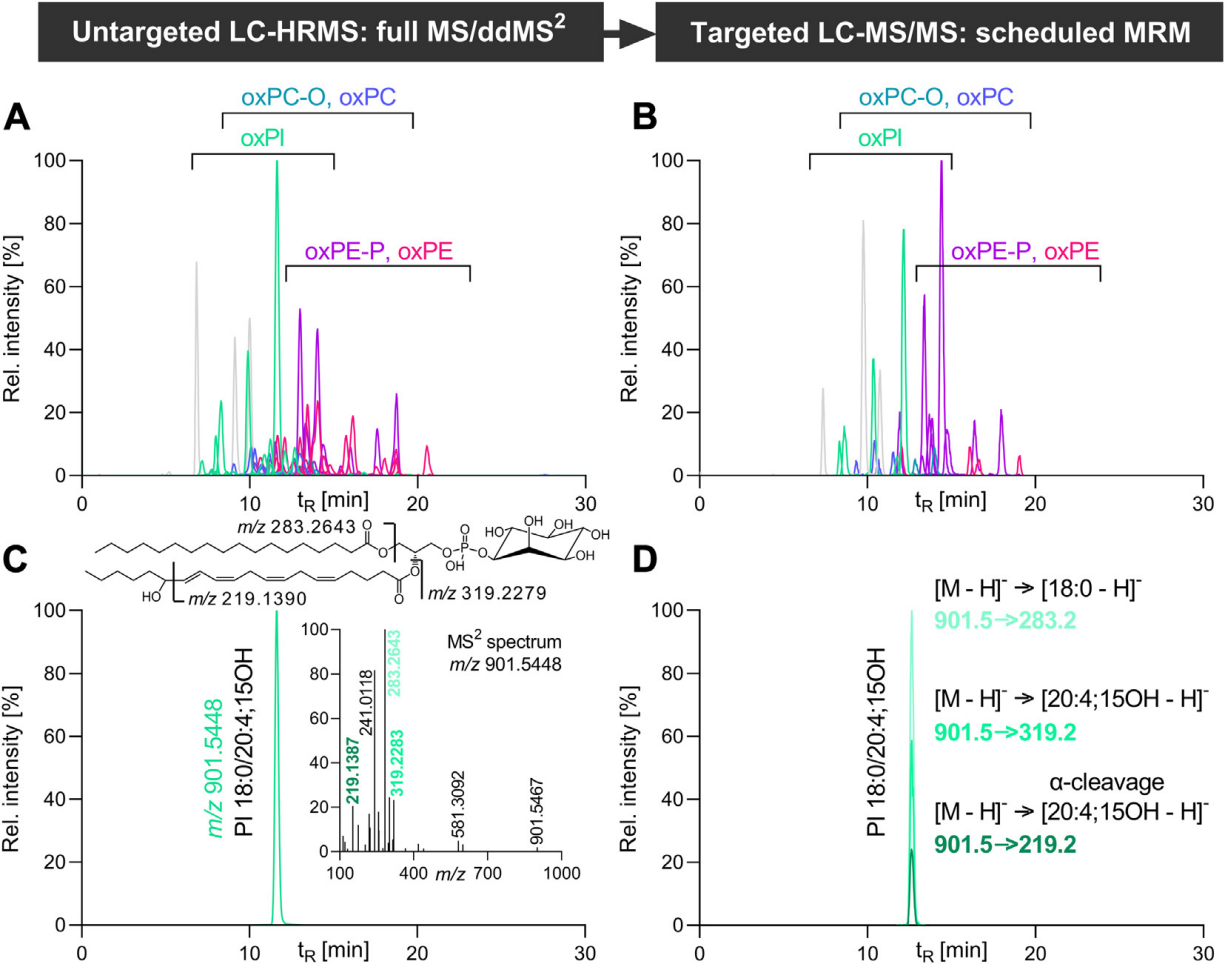
Development of a targeted LC-MS/MS method based on an untargeted LC-HRMS method. Shown is the detection in ESI(−) mode of PLs bearing hydroxy-PUFAs in 15-LOX-2-overexpressing HEK293T_15-LOX-2 cells (200 ng/ml doxycycline, 24 h): (A) untargeted LC-HRMS in full MS/ddMS^2^ mode (Q Exactive HF) and (B) by targeted LC-MS/MS in scheduled MRM (QTRAP 6500+). C: HRMS-extracted ion chromatogram as well as MS^2^ spectrum. D: MS/MS MRM chromatograms of PI 18:0/20:4;15OH. MRM, multiple reaction monitoring.

Therefore, we developed a targeted LC-MS/MS method for the direct analysis of PLs bearing hydroxy-PUFAs in HEK293T cells based on an established untargeted LC-HRMS method (48) (Fig. 2): OxPLs were screened in full MS/ddMS^2^ mode in lipid cell extracts and characterized based on MS^2^ spectra and retention time as described (48) (Fig. 2A-C). Notably, no oxylipins were found esterified in PLs with a sphingosine backbone, that is, sphingomyelin. Untargeted LC-HRMS detected the hydroxy-PUFAs bound to PI, PC, PE, PC-P (plasmalogen PC), ether PC (PC-O), and plasmalogen PE (PE-P) lipid classes but none in lyso-PL, PS, or PG (Fig. 2A). The detected PL bear 16:0, 16:1, 18:0, and 18:1 in the *sn*-1 position and 18:2;13OH, 20:4;15OH, 20:5;15OH, 22:4;17OH, 22:5;17OH, and 22:6;17OH in the *sn*-2 position. The most relevant oxPL species were included in the targeted LC-MS/MS method. Three mass transitions were selected to monitor product ions corresponding to *i*) the saturated FA, *ii*) the oxPUFA, and *iii*) the characteristic product ion resulting from the α-cleavage of the oxPUFA at the hydroxy group (Fig. 2D): For example, PI 18:0/20:4;15OH was detected with the mass transitions *m/z* 901.5 → 319.2, *m/z* 901.5 → 283.2, and *m/z* 901.5 → 219.2. In total, 67 oxPL species from six different PL classes (i.e., PI, PC, PE, PC-P, PC-O, and PE-P) were included in the targeted multiple reaction monitoring method (Table 1, Supplemental Table S1).

The new targeted method was characterized and validated (Supplemental Fig. S2–S6, and Supplemental Tables S2, S3, S5, S6, S7). In brief, the sensitivity was good with an LLOQ ranging from 30 to 125 fmol on column, and a linear range of 30–5,000 fmol. PLs bearing short fatty acyl chains (e.g., PE 12:0/13:0) were used as IS (Supplemental Fig S2A, B). Liquid-liquid extraction using 0.38 mg cellular protein resulted in sensitive detection of oxPL formed in HEK293T_15-LOX-2 with minimal ion suppression affecting oxPL and IS signals (Supplemental Fig. S3). The method showed high extraction recoveries of IS and oxPL spiked to cell samples >80% (Supplemental Figs. S24, S4), a good intraday/interday accuracy (66 − 112%) and precision (100 ± 15%) of spiked oxPLs were obtained (Supplemental Table S7), with a few exceptions that can be resolved with 1:10 dilution of the oxPL extract (Supplemental Figs. S5B, Fig. S6). Detailed information on method development and validation as well as comparison of its performance to earlier described methods can be found in the supplemental data.

**Fig. 3:**
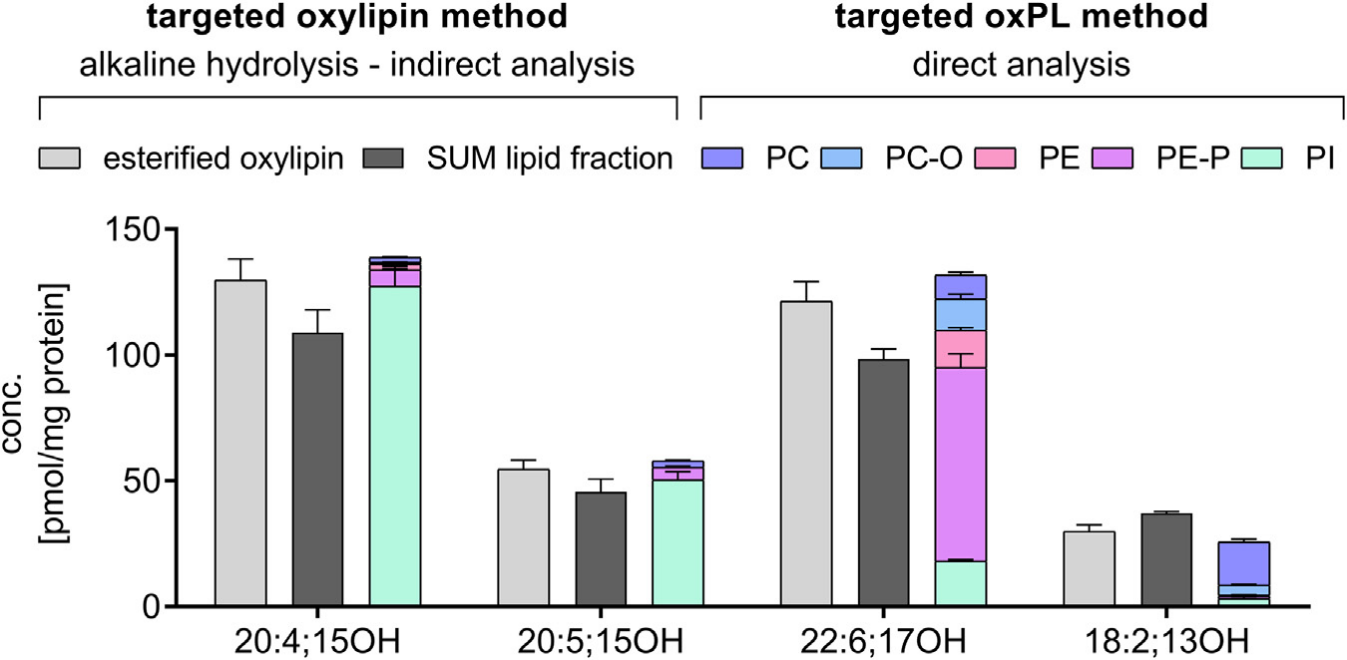
Targeted analysis of PLs bearing hydroxy-PUFAs leads to similar concentration as targeted esterified oxylipin analysis. Esterified oxylipins were quantified in 15-LOX-2-overexpressing 5 × 10^6^ HEK293T_15-LOX-2 cells either indirectly following alkaline hydrolysis or directly as intact oxPL by targeted LC-MS/MS methods. Shown is the comparison of the results. (Light gray bar) esterified oxylipins determined as total—nonesterified oxylipins. (Dark gray bar) sum of esterified oxylipins in the HILIC-separated lipid fractions. (Colored bars) sums of individual oxPL species of each lipid class. Shown is the mean concentration ± SD per milligram cellular protein (n = 3).

Applying the developed targeted oxPL LC-MS/MS method for the analysis of HEK293T cells, the resulting concentrations (sum of individual oxPL species) were remarkably consistent with the indirect quantification of esterified oxylipins following alkaline hydrolysis (with and without HILIC-based SPE fractionation). This is shown in Figure 3 exemplarily for 15-LOX-2-overexpressing cells: The differences between (the sum of) concentrations determined by targeted quantification of oxPL were <14% compared with the targeted oxylipin method, the current gold standard for esterified oxylipin analyses. This underlines that the new targeted oxPL method covers all relevant oxylipin-bearing species, and this also demonstrates the accuracy of the method. Thus, esterified 15-LOX-2 products bound in PL can be accurately quantified as individual oxPL species by the developed targeted method.

### Incorporation of oxylipins into distinct glycero-PL species

**Fig. 4:**
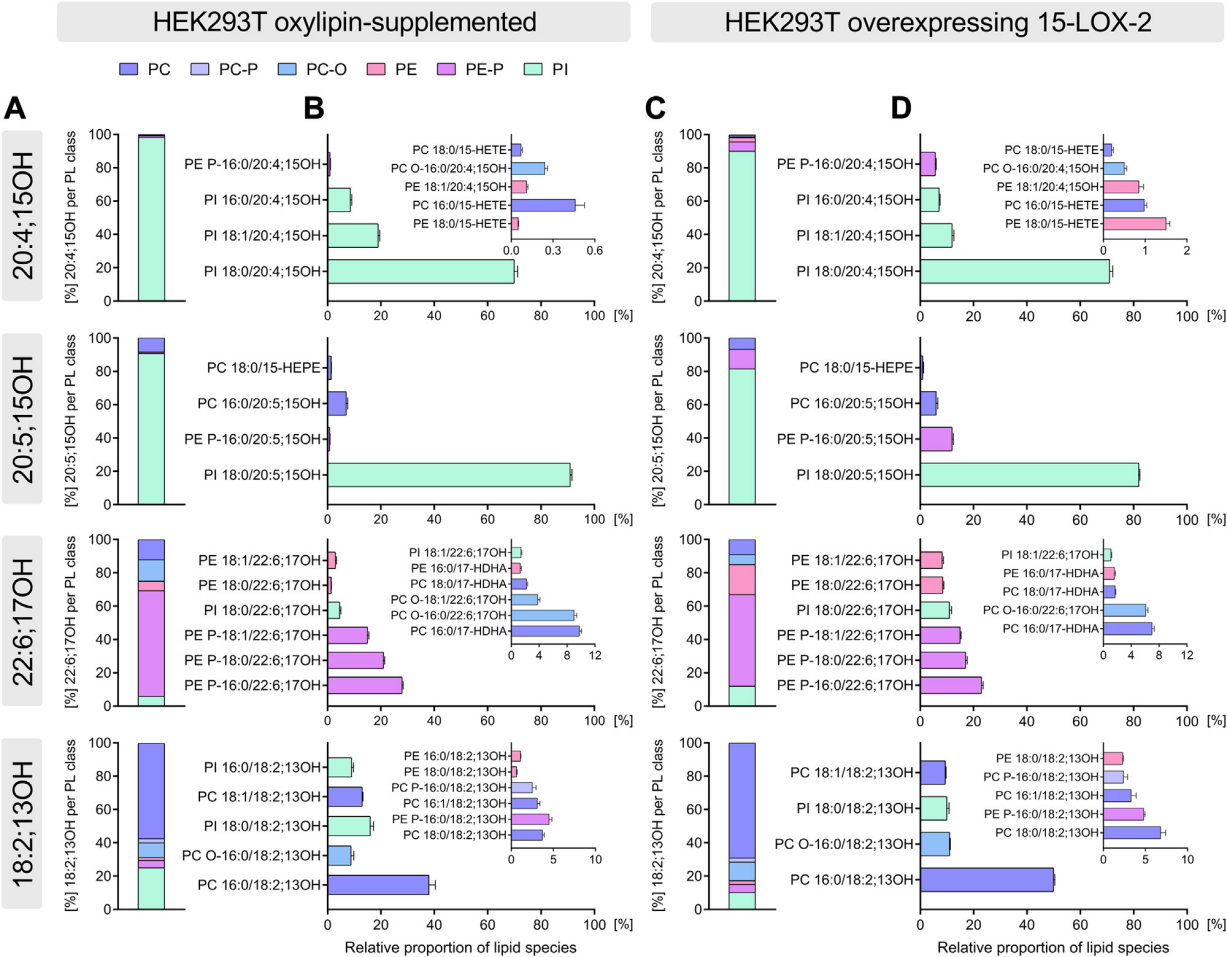
15-LOX-2 products of PUFAs are esterified to distinct PL classes and specific molecular PL species following supplementation and in 15-LOX-2-overexpressing cells. Around 5 × 10^6^ cells were used per experiment. Left: HEK293T cells supplemented with either 15(*S*)-HETE, 15(*S*)-HEPE, 17(*S*)-HDHA, or 13(*S*)-HODE (300 nM, 2 h). Right: Endogenous formation of oxPL elicited by overexpression of 15-LOX-2 in HEK293T_15-LOX-2 cells (200 ng/ml doxycycline, 24 h). Shown is the relative distribution of 20:4;15OH, 20:5;15OH, 22:6;17OH, and 18:2;13OH (A and C) in PL classes and (B and D) for the PL molecular species. For each oxylipin, the percentage of the sum of all oxPL quantified by targeted LC-MS/MS was calculated. Mean concentration ± SD per milligram cellular protein (n = 3) can be found in Supplemental Table S4.

Using the developed targeted LC-MS/MS method, we could show that esterified 15-LOX-2 products occur in a few PL species (Fig. 4): The oxylipins were always found in *sn*-2 position of the PL species (Fig. 4B, D). The fatty acyls at *sn*-1 were dominantly 16:0 (PC, PE-P, and PC-O) and 18:0 (PI and PE). Analysis of oxPL in HEK293T cells following supplementation or overexpression of 15-LOX-2 showed a comparable distribution of oxylipins both in the PL classes (Fig. 4A, C) and across PL species (Fig. 4B, D), except for PE as shown for 15-HETE and 15-HEPE (Fig. 1B): In 15-LOX-2-overexpressing cells (Fig. 4D), 5.7% of 20:4;15OH and 12% of 20:5;15OH were incorporated into PE-P bearing 16:0 at the *sn-*1 position, whereas <1% of supplemented 20:4;15OH and 20:5;15OH were found in these lipids. 15-LOX-2 can oxidize both free FA but also PUFA esterified in PL (3, 5). Following incubation of cell homogenates with exogenous 15-LOX-2 (Supplemental Fig. S8), 61% of 20:4;15OH was bound to PI species compared with at least 90% for 20:4;15OH in supplemented and 15-LOX-2-overexpressing cells. Almost half of the 20:5;15OH formed by PL oxidation by 15-LOX-2 was incorporated into PE-P species, whereas at least 80% of supplemented and endogenously formed 20:5;15OH was esterified into PI.

A notable specificity of oxylipin incorporation toward certain PL species was observed (Fig. 4B, D), both in supplemented and 15-LOX-2-overexpressing cells: While 20:4;15OH (15-HETE) and 20:5;15OH (15-HEPE) showed a narrow distribution between few PI species, 22:6;17OH (17-HDHA) and 18:2;13OH (13-HODE) were more evenly distributed across the PL classes and species. 20:4;15OH was predominantly bound to the species PI 18:0/20:4;15OH with 71 ± 1% in 15-LOX-2-overexpressing cells (Fig. 4D). 20:4;15OH was detected in notable amounts in PI 18:1/20:4;15OH and PI 16:0/20:4;15OH, with 12.4 ± 0.6% and 7.1 ± 0.4%, respectively. A minor part of 20:4;15OH was detected in PE-P 16:0/20:4;15OH with 5.7 ± 0.3%, whereas the six remaining PC and PE species detected represented each <1.5%. Similar to 20:4;15OH, the vast majority of 20:5;15OH was found in PI 18:0/20:5;15OH accounting for 81.6 ± 0.4%. At lower amount, 20:5;15OH was found in PE P-16:0/20:4;15OH and PC 16:0/20:5;15OH species, with 11.7 ± 0.4% and 5.9 ± 0.6%, respectively. Direct oxPL analysis showed that 22:6;17OH is predominantly esterified to PE-P: More than 50% was detected among the three species PE P-16:0/22:6;17OH (23%), PE P-18:0/22:6;17OH (17%), and PE P-18:1/22:6;17OH (15%) in cells overexpressing 15-LOX-2. The second most abundant PL class was PI, with the species PI 18:0/22:6;17OH accounting for 11 ± 1%. Around 30% of 22:6;17OH was distributed quasiequally between four lipid species, that is, PE bearing 18:0 and 18:1, and PC(-O) bearing 16:0. 18:2;13OH was detected in four PC species bearing 16:0, 16:1, 18:0, and 18:1 in the *sn*-1 position but was predominantly bound to PC 16:0/18:2;13OH with 49.6 ± 0.4%. Each of the three species, PC O-16:0/18;13OH, PI 18:0/18;13OH, and PC 18:1/18;13OH, accounted for ∼10% of esterified 18:2;13OH. The last 20% of 18:2;13OH was distributed between the six lowest-concentration PC(-O) and PE(-P) species. The differences observed in oxylipin distribution across PL species seem not to depend on the concentration: Among the analyzed oxylipins in cells overexpressing 15-LOX-2 (Fig. 3), 18:2;13OH is the least abundant with 29.9 ± 2.5 pmol/mg, whereas 20:5;15OH is nearly twice as abundant at 54.6 ± 3.5 pmol/mg and exhibits a restricted distribution between only four oxPL species (Fig. 4D).

## Discussion

Oxylipins were found to be distinctly incorporated into both PL classes and molecular species (Fig. 1B, Fig. 4). Supplementation and endogenous formation of oxylipins led to a comparable incorporation pattern, whereas direct oxidation of cell PL by exogenously added 15-LOX-2 was different (Supplemental Fig. S8). These results indicate that for the formation of oxPL in the 15-LOX-2-overexpressing cells, FAs are first released, the nonesterified FA is oxidized, and then incorporated into lyso-PL and not formed by direct oxidation of intact PL by the LOX enzyme. The observed distinct incorporation of oxylipins into the membranes (Fig. 1B, Fig. 4) thus depends on the specificity of the enzymes generating the PL during the Lands’ remodeling cycle. This involves the acyl-CoA synthetases, that is, the different long-chain acyl-CoA synthetases (ACSL) and lyso-PL acyl transferase (LPLAT) (53, 54). Different ACSL isoforms (i.e., ACSL-1, -3, -4, -5, and -6) accept 15-, 12-, and 5-HETE as a substrate (55). Thus, the LPLAT-catalyzed transfer of activated acyl-CoA into lyso-PL seems to cause this distinct incorporation. Fourteen LPLATs have been identified exhibiting pronounced differences in substrate preferences and lyso-PL enzymatic activities (53, 56, 57, 58, 59, 60). For example, while LPLAT12 (LPCAT3) incorporates 18:2- and 20:4-CoAs into lyso-PC, lyso-PE, and lyso-PS (58), LPLAT13 (MBOAT2) and LPLAT14 (MBOAT1) both transfer 22:6-CoA into lyso-PE (and lyso-PC for LPLAT13) (60). Moreover, LPLAT11 (MBOAT7) transfers 20:4- and 20:5-CoAs predominantly into lyso-PI (56). Inhibition of LPLAT12 using 10 µM (*R*)-HTS-3 reduced the formation of PC and PE species bearing 12-HETE, but not those containing 11- or 15-HETE, in thrombin-activated platelets (61). These results would indicate that LPLAT12 exhibits selectivity toward oxylipin positional isomers. The almost exclusive incorporation of 20:4;15OH and 20:4;15OH into PI observed in this study thus might result from LPLAT11. In contrast, the esterification of 22:6;17OH is likely catalyzed by other LPLATs, for example, LPLAT13 or LPLAT14. Similarly, incorporation of 18:2;13OH into lyso-PC might be catalyzed by LPLAT12.

Preferred incorporation of 15-HETE into PI has been previously described following supplementation of labeled [^3^H]/[^14^C]15-HETE (0.1–1 μM, 20–120 min) in bovine pulmonary arterial endothelial cells (30), human primary neutrophils (31), mouse macrophage-like RAW 5774.2 cell line (32), human tracheal epithelial cells (29), and Madin-Darby canine kidney cells (27). Using radioactively labeled oxylipins, these studies showed by TLC that between 69% and 86% of labeled 15-HETE was incorporated into PI, and in smaller amounts into PC and PE, ranging from 4.9% to 21% for PC plus PE. Similarly, favored incorporation of [^14^C]13-HODE into PC was shown: Cho *et al*. (28) reported by TLC that 58% of [^14^C]13-HODE was incorporated into PC, 23% into PI, 12% into PS, and 6.8% into PE following supplementation in the epidermis (1.6 μM, 6 h). In this study, consistent incorporation patterns of 20:4;15OH and 18:3;13OH into PL classes were found following supplementation in HEK293T cells (Fig. 1B, Fig. 4A, B). Specific incorporation of 15-HETE into PI bearing 18:0 at the *sn*-1 position was reported in two studies. Legrand *et al*. identified [^3^H]15(*S*)-HETE in PI bearing 18:0 or 18:1 by GC-MS following separation by TLC in supplemented endothelial cells (1 μM, 2 h). Interestingly, more than 92% of 15-HETE formed via 15-LOX-1 in activated human monocytes was incorporated into PE due to direct oxidation of PL, while exogenously added 15-HETE was esterified but not detected in PL (33). In our previous work, nonlabeled 20:4;15OH was identified exclusively into PI species containing 18:0, 18:1, or 16:0 in 15-HETE-supplemented HEK293 cells (2 μM, 2 h) (48). Here, targeted LC-MS/MS not only supported this distinct incorporation of 20:4;15OH into these three PI species (Fig. 4B, D) but also enabled the detection of low concentrated PC and PE species bearing 20:4;15OH. Moreover, we could also show a similar specific incorporation of 20:5;15OH into PI bearing 18:0. 22:6;17OH and 18:2;13OH were found to be more evenly distributed into PE-P and PC species, respectively, but 10% of these oxylipins were as well bound to PI bearing 18:0 (Fig. 4D).

Overall, these findings could be of high importance as PI species are involved in two main regulatory pathways, which impact cell growth and signaling (62, 63, 64, 65). In the PI-cycle pathway, PI is converted to PI 4,5-bisphosphate (PIP_2_) by a two-step reaction. Binding of an agonist to a Gq-coupled receptor leads to the activation of phospholipase C, which cleaves PIP_2_ into DG and inositol 1,4,5-trisphosphate (62, 63). Both act as second messengers. Inositol 1,4,5-trisphosphate activates protein kinase C regulating, for example, the production of proinflammatory cytokines (66). In the protein kinase B (Akt/PKB) signaling pathway, PIP_2_ is phosphorylated to PI 3,4,5-trisphosphate via phosphoinositide 3-kinases. PI 3,4,5-trisphosphate then recruits Akt/PKB to the plasma membrane enabling its phosphorylation by phosphoinositide-dependent kinase-1, controlling, for example, cell proliferation via Akt/PKB and mammalian target of rapamycin (64). In this context, specific incorporation into PI of 15-HETE and 15-HEPE could play a key role in the regulation of these pathways. Indeed, previous studies described that [^3^H]/[^14^C]15-HETE caused an elevation of labeled 15-HETE-DG in bovine pulmonary endothelial cells exposed to bradykinin (30), and in epithelial cells, treatment with platelet-activated factor was associated with a selective decrease in [^3^H]15-HETE-PI (26). In the same study, [^14^C]15-HETE-DG was able to stimulate protein kinase C-α (26). Setty *et al*. (67) reported that 15-HETE caused an elevation of cellular DG as well as a stimulation of endothelial cells’ DNA synthesis and cell growth. Here, we show that the 15-LOX products of DHA and linoleic acid are not incorporated into PI. Thus, a shift in the FA pattern could alter PI signaling. It is well known that intake of long-chain n3-PUFAs leads to a shift in the FA composition, thereby increasing n3-PUFAs (largely DHA) and decreasing n6-PUFAs (largely ARA) (68, 69, 70). The formation and occurrence of hydroxy-PUFAs correlates with the concentration of the substrate (71, 72), and 15-LOX even prefers DHA as a substrate compared with ARA (7). As we show here, hydroxy-PUFAs occur exclusively esterified and dominantly in PL (Fig. 1B), the diet directly seems to also modify the oxylipin pattern and thus the PL membrane composition. Therefore, a diet rich in long-chain n3-PUFA increasing 17-HDHA via 15-LOX while decreasing 15-HETE might influence PI signaling pathway(s), which could contribute to the biological effects of an n3-PUFA-rich diet (73, 74, 75). Additionally, several studies demonstrate that 15-HETE and 15-HEPE have anti-inflammatory effects (17). Importantly, the preferential incorporation of 15-HETE into PI is not limited to the supplemented or 15-LOX-2-overexpressing HEK293T cells used in this study but has also been consistently observed in a variety of other cell types following supplementation (26, 27, 29, 30, 31, 32)—highlighting the physiological relevance of this incorporation. With the modification of PI-based signaling, we suggest here a new pathway that may explain the effects of n3-PUFAs and 15-LOX products, warranting further investigation.

## Conclusion

Combined analysis of esterified oxylipins enabled the characterization in which PL classes and molecular PL species 15-LOX-2 oxylipin products are located in HEK293T cells. Indirect quantification of esterified oxylipins in lipid fractions identified the specific PL classes in which 15-LOX-2 oxylipin products are located at baseline, following supplementation, and in 15-LOX-2-overexpressing cells. Targeted LC-MS/MS quantification of PLs bearing hydroxy-PUFAs enabled to pinpoint in which molecular species and *sn*-1/*sn*-2 position oxylipins are esterified following supplementation and endogenous formation. The vast majority of 15-HETE and 15-HEPE was incorporated into distinct PI species with more than 70% of 20:4;15OH and 80% of 20:5;15OH esterified in the *sn*-2 position of PI bearing 18:0. On the opposite, 17-HDHA and 13-HODE were more evenly distributed into PL classes and species. 17-HDHA was predominantly found in PE, particularly PE-P species bearing 16:0, 18:0, and 18:1 in the *sn*-1 position. Most of 13-HODE was found in PC with a marked abundance of PC 16:0/18:2;13OH at 50%, whereas the other half was distributed mainly between PC(-O) and PI species. For these two oxylipins, the second most abundant PL class was PI, specifically PI bearing 18:0. Based on this accurate quantification using two orthogonal approaches, a distinct incorporation of 15-LOX-formed oxylipins both into PL classes/species was shown. The associated biological effects remain to be uncovered and may explain the anti-inflammatory effects of n3-PUFAs and 15-LOX by altering PI-based signaling pathways.

## Data availability

All data are contained within the article or supplemental data.

## Supplemental data

This article contains supplemental data (24, 46, 49, 34, 35, 76, 77, 78, 79).

## Conflict of interest

The authors declare that they have no conflicts of interest with the contents of this article.
