# Supplementary material for: Substrate-dependent incorporation of 15-lipoxygenase products in glycerophospholipids: 15-HETE and 15-HEPE in PI, 17-HDHA in plasmalogen PE, and 13-HODE in PC
Source: J Lipid Res. 2025 Jun 14;66(7):100841. doi: 10.1016/j.jlr.2025.100841 (PMC12274753; doi:10.1016/j.jlr.2025.100841)
Supplement: Supplementary Material 1 [file mmc1.pdf]

Substrate-dependent incorporation of 15-lipoxygenase products in glycerophospholipids: 15-HETE and 15-HEPE in PI, 17-HDHA in plasmalogen PE, and 13-HODE in PC

Supplemental data

Laura Carpanedo[1], Luca Marcel Wende[1], Bjarne Goebel[2], Ann-Kathrin Häfner[2], Michel André Chromik[1], Nadja Kampschulte[1], Dieter Steinhilber[2], and Nils Helge Schebb[1*]

[1]Chair of Food Chemistry, Faculty of Mathematics and Natural Sciences, University of Wuppertal, Wuppertal, Germany,

[2]Institute of Pharmaceutical Chemistry, Goethe University Frankfurt, Frankfurt, Germany

Materials and methods

Targeted LC-MS/MS Proteomics

Sample preparation was carried out as described (1). In brief, the cell pellet obtained after the protein precipitation was re-dissolved in 5% w/v sodium deoxycholate containing 1% protease inhibitor mix (39102.02, SERVA Electrophoresis GmbH, Heidelberg, Germany), sonicated, and centrifuged (4 °C, 10 min, 15,000 × *g*). A solution containing 500 µg total protein was used for analysis. Proteins were precipitated and washed with four volumes of ice-cold acetone. Dried pellets were reconstituted in 6 M urea to a final concentration of 5 mg/mL. The disulfide bridges were reduced for 1 h with 200 mM dithiothreitol (in 50 mM NH_4HCO_3), and the resulting free sulfhydryl groups were alkylated for 1 h with 200 mM iodoacetamide (in 50 mM NH_4HCO_3).

Tryptic digestion using >6.000 U/g trypsin from porcine pancreas (Merck 37286.03) was performed for 15 h at 37 °C (pH≈7.8). The digestion was stopped by the addition of concentrated acetic acid, reducing the pH to 3–4. Heavy labeled peptides (lys: U-$^{13}C_6$; U-$^{15}N_2$; arg: U-$^{13}C_6$; U-$^{15}N_4$) serving as IS were added. Following centrifugation, peptides were extracted by SPE (using Strata-X 33 µm polymeric reversed phase material, Phenomenex LTD, Aschaffenburg, Germany) and evaporated to dryness in a vacuum centrifuge. Residues were reconstituted in 15% ACN containing 0.1% acetic acid and centrifuged (4°C, 10 min, 15,000 x *g*). Clear supernatants were analyzed by targeted LC-MS/MS proteomics analysis.

Targeted LC-MS/MS analysis of peptides was carried out using a 1290 Infinity II (Agilent, Waldbronn, Germany) LC system coupled to a QTRAP 6500+ mass spectrometer (Sciex, Darmstadt, Germany) as described (1). Chromatographic separation was carried out on a ZORBAX Eclipse Plus C18 column (2.1 × 150 mm, 1.8 µm, 95 Å; Agilent) equipped with a guard column (2.1 × 2 mm, 1.8 µm) at 40 °C. A binary gradient was used with eluent A (H_2O/acetonitrile, 95:5, v/v) and eluent B (H_2O/acetonitrile, 5:95, v/v/v), both containing 0.1% acetic acid. Peptides were separated using the following gradient with a flow rate of 300 µL/min: 0–1.0 min 0% B; 1.0–30.5 min 0–35% B; 30.5–30.6 min 35–100% B; 30.6–33.5 min 100% B; 33.5–33.7 min 100–0% B; 33.7–36 min 0% B. MS detection of peptides was performed in scheduled MRM mode following positive ESI. Peptides were quantified using external calibrations with IS, and concentrations were normalized based on the amount of cellular protein.

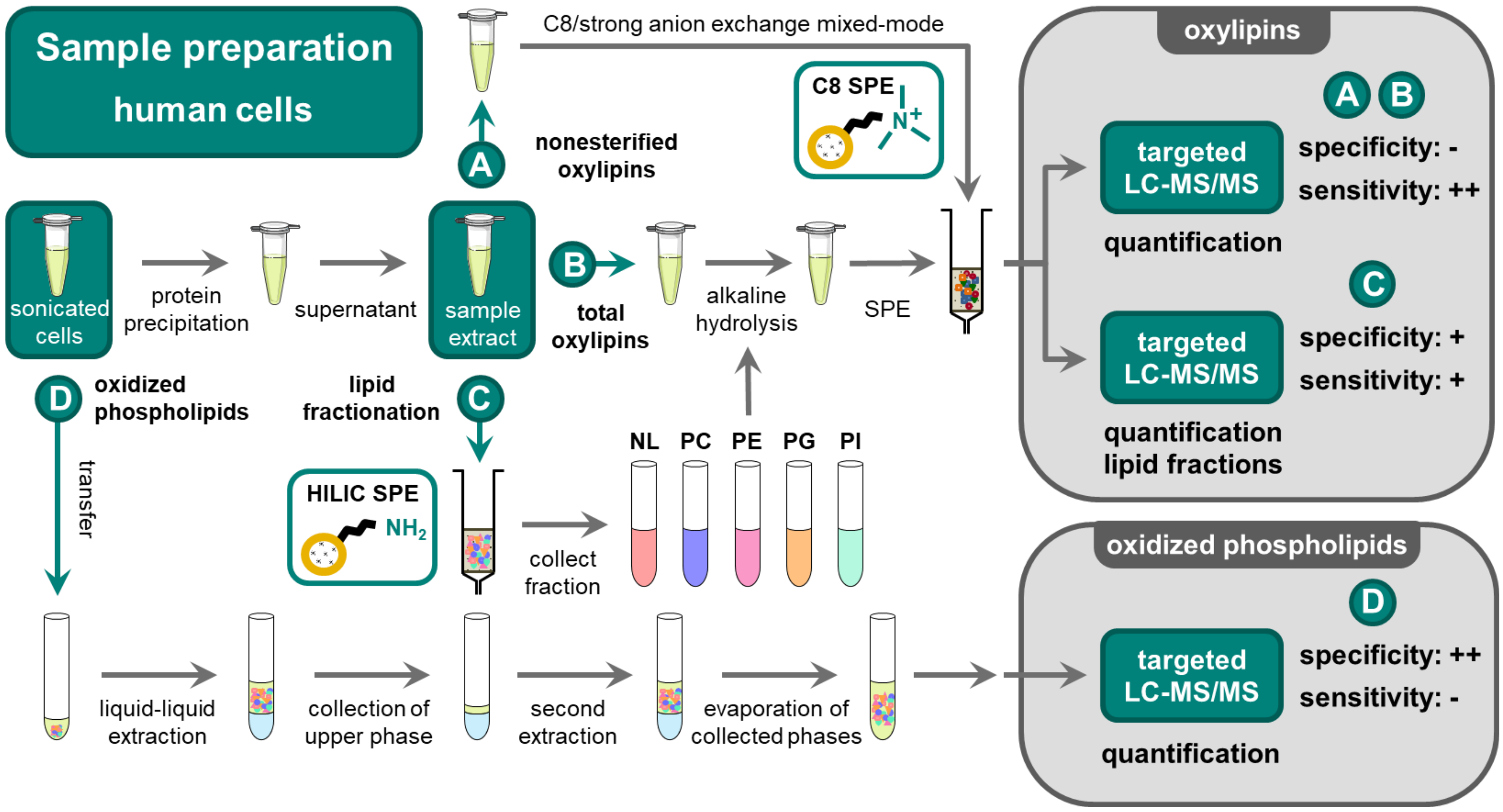

Fig. S1: Workflow of comprehensive oxylipin analysis. In (A) nonesterified oxylipins are analyzed directly. In (B) total oxylipins are analyzed and quantified as nonesterified oxylipins following hydrolysis. (C) To quantify oxylipins bound in different PLs, the lipids were fractionated by HILIC-SPE prior to hydrolysis, followed by the quantification of esterified oxylipins in each fraction. In (D) PLs bearing oxylipins are analyzed directly.

Development and characterization of a targeted LC-MS/MS method for glycero-phospholipids bearing oxylipins

Method performance

Quantification of the oxPLs was carried out based on the analyte to corresponding IS area ratio using linear calibration. OxPC and oxPE species were quantified using identical standards or, when not available, standards of the same lipid class and similar fatty acyl chain composition Table S3. OxPI species were quantified using OxPC species standards with similar fatty acyl chain composition. PLs bearing 18:2;13OH were quantified using PL standards containing 20:4;15OH, while PLs bearing 22:5;17OH were quantified using PL standards containing 22:6;17OH. For oxPL species whose standard was not available, the relative concentration was determined using selected ion monitoring (SIM)/MRM measurements of both the lipid extracts and standard solutions, assuming similar ionization efficiency (2). Based on that, a factor of the MRM signal was calculated for each analyte and the corresponding compound used as standard (Table S3). IS was assigned for each oxPL species based on the polar head group: PI 12:0/13:0 for oxPI species, PC 12:0/13:0 for oxPC species, and PE 12:0/13:0 for oxPE species. For oxPI quantification, PI 12:0/13:0 was used as IS to calculate the area ratio for the calibration (Table S2).

The sensitivity of the method was evaluated by determining LOD and LLOQ based on the signal-to-noise ratio (S/N, peak to peak). The concentration yielding an S/N $\geq$ 3 was defined as LOD. Analytes showed an LOD ranging from 7.5 to 30 fmol (0.59 to 4.8 pg) on column, i.e., 1.5 to 12 nM, (Table S2). The concentration with a S/N $\geq$ 5 and an accuracy of 80 – 120% within the linear calibration was defined as LLOQ and set as the lowest concentration of the linear calibration. All oxPLs were detected with an LLOQ in the same order of magnitude from 30 to 125 fmol (2.36 to 10 pg) on column, i.e., 6 to 25 nM in the injected solution. Few studies have reported information about LOD, LLOQ, and the linear range of oxPL species. Linearity was assessed using standard solutions covering a concentration range from 0.75 to 1000 nM (30 – 5000 fmol on column).

Few methods also investigated oxPLs using a triple quadrupole mass spectrometer: Slatter *et al.* established a targeted method for 111 oxPL species in human platelets (3), Aoyagi *et al.* quantified 20 oxPL species in mouse peritoneal macrophages (4), and Nakanishi *et al*. analyzed 44 PC species bearing hydroxy-PUFAs, hydroperoxy-PUFAs, aldehyde bearing PUFAs, and carboxylated PUFAs (5). If provided, these methods had a similar sensitivity and linearity as the method developed here, but none of them includes all the important PLs bearing hydroxy-PUFAs as in the present study. Aoyagi *et al.* found a LOD of 10 fmol for oxPCs and oxPEs using a QTRAP6500 mass spectrometer and a linear range of 10 – 500 fmol on column (4). Using a QTRAP4000 mass spectrometer, Nakanishi *et al.* reported a linear range of 50 – 100,000 fmol on column (5).

Choice of internal standards

IS are key for accurate quantification as they compensate for sample losses during extraction, ion suppression, correct for instrumental variability, and enhance the reproducibility. In untargeted lipidomics, at least one isotopically labeled IS is employed per lipid class. However, no deuterium (^{2}H, D)- or ^{13}C-labeled oxPLs are commercially available. Therefore, unoxidized PLs – either deuterium-labeled or containing odd-carbon fatty acyl chains – were evaluated regarding their suitability as IS (Fig. S2).

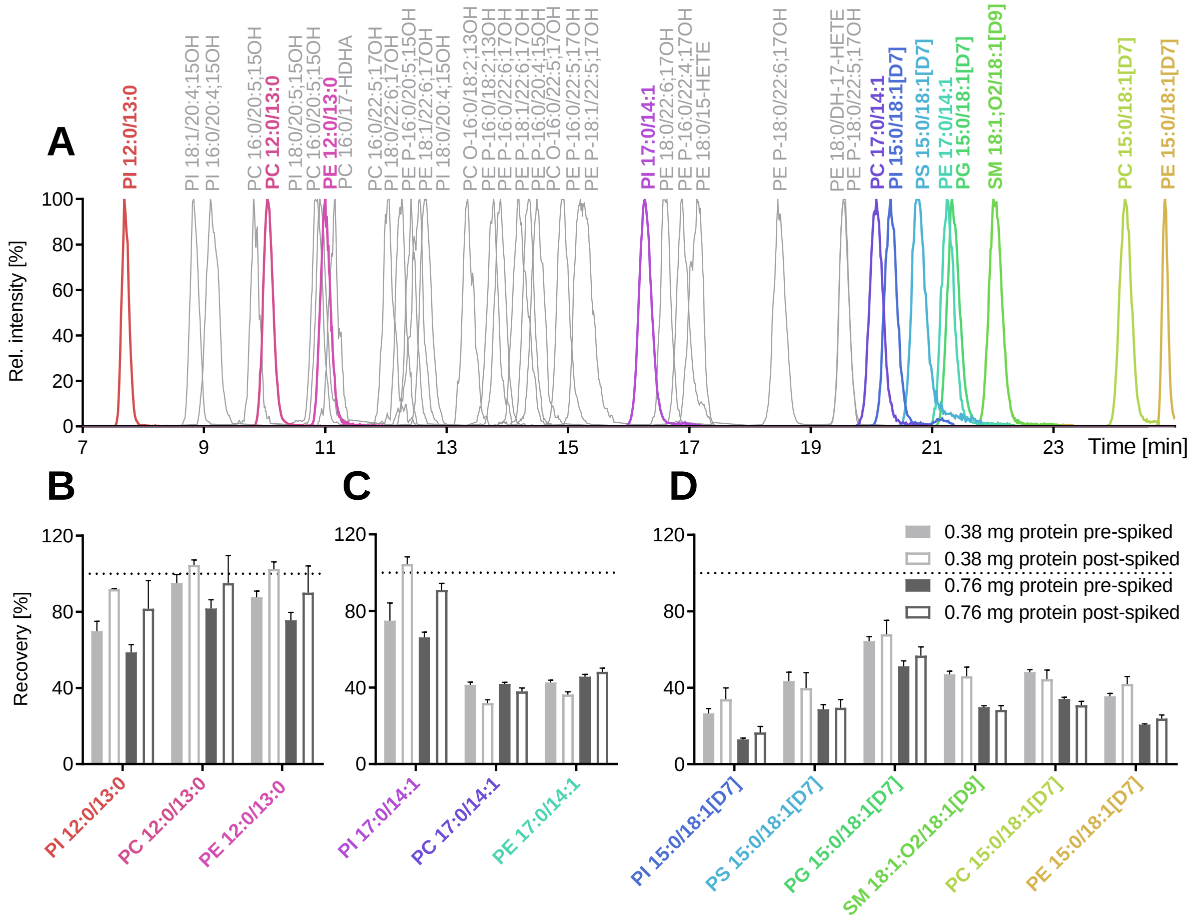

Fig. S2: Choice of IS. (A**)** Elution order of possible ISs compared to PLs bearing hydroxy-PUFAs detected in HEK293T_15-LOX-2 cells overexpressing 15-LOX-2. In (B, C, and D**)** the recoveries of the ISs (2.5 pmol of each) added before or after sample preparation (pre- or post-spiked) were evaluated using different amounts of sample (0.38 mg protein or 0.76 mg protein). Shown is the mean ± SD (n = 3).

Comparison of recoveries of IS spiked to cell samples added prior to or after post-extraction showed acceptable losses of less than 20% during extraction for all ISs tested (Fig. S2B, C, D). Adding the ISs after sample preparation revealed relevant ion suppression effects for those potential ISs (i.e., deuterium-labeled PLs as well as PC 17:0/14:1 and PE 17:0/14:1) eluting after the elution window of oxPLs (i.e., 20 min), with over 50% signal suppression (Fig. S2A, C, D).

Therefore, these compounds are not suitable as IS. Within the early elution window of oxPLs, from 7 to 11 min, ion suppression effects were minor (Fig. S2A, B): Using 0.76 mg of protein for LLE, the signals of PI, PC and PE bearing 12:0 and 13:0 were partially suppressed (less than 20%) while no matrix effects were observed using 0.38 mg of protein. Thus, the three unoxidized PLs, i.e., PI, PC, and PE bearing 12:0 and 13:0, were chosen as IS. Moreover, the amount of sample was set to 0.38 mg protein for extraction because no apparent ion suppression effects were observed.

Our results showed that strong ion suppression effects occur after the elution window of oxPLs, while minor matrix effects are observed in the early elution window of oxPLs. This indicates that PLs with short fatty acyl chains are suitable ISs for oxPLs. This is in line with previous studies where PE 14:0/14:0 was used as IS for the quantification of oxPLs in human platelets (3) and mice organs (6). Also, PC 14:0/14:0 was used for the quantification of oxPCs in human serum (7). Aoyagi *et al.* used PI, PS, PG, PC, and PE, all bearing 17:0 and 14:1 as IS for the analysis of oxPLs in primary mouse peritoneal macrophages (4), but did not provide information about extraction recovery. In our hands, the signal of both PC 17:0/14:1 and PE 17:0/14:1 is partially suppressed by the HEK293T cell matrix and thus are not suitable for the tested matrix. Because no isotopically labeled oxPL are available, the selection of PLs as IS requires caution, and their extraction recovery should be evaluated in the matrix using prior- and post-spiking experiments as described here.

Effect of sample amount on the concentration of endogenous oxidized glycerol-PL

Investigation of recoveries of ISs added at the end of the sample preparation allowed the selection of suitable ISs for the analysis of oxPLs (see above). Here we found no or minimal interference at a sample amount of 0.38 mg protein. In the next step, the effect of the sample amount was further characterized based on the determined apparent concentration of endogenous oxPLs (Fig. S3).

Analysis of samples containing 0.38 mg protein allowed a sensitive detection of oxPLs in 15-LOX-2 overexpressing cells: A total of 40 oxPL species were quantified, while 0.76 mg protein led to the additional detection of 6 oxPL species. However, considerably fewer oxPLs were detected when lower amounts of protein were used, with 30 oxPL species quantified at 0.19 mg protein and 20 oxPL species at 0.076 mg protein.

Using different cellular protein amounts for LLE, consistent concentrations were obtained for 16 of the 20 detected oxPLs, with less than 18% difference compared to the value obtained with 0.38 mg protein (Fig. S3A). For PE 18:0/22:6;17OH and PE 18:0/15-HETE, the use of 0.57 mg protein or more leads to deviations by ion suppression/enhancement effects, indicating that 0.38 mg protein or less should be analyzed. An unacceptable variation in concentrations was found for the last-eluting analyte, i.e., PE P-18:0/22:5;17OH, probably caused by ion enhancement effects.

The signals of ISs were partially suppressed when 0.57 mg and 0.76 mg protein were used for extraction (Fig. S3B): Apparent recoveries of PI 12:0/13:0, PC 12:0/13:0, and PE 12:0/13:0 were 37%, 61%, and 76%, respectively, when 0.76 mg protein was used for LLE. However, good recoveries of 62%, 90%, and 104% were achieved when 0.38 mg protein was used for LLE, as previously shown (Fig. S2B).

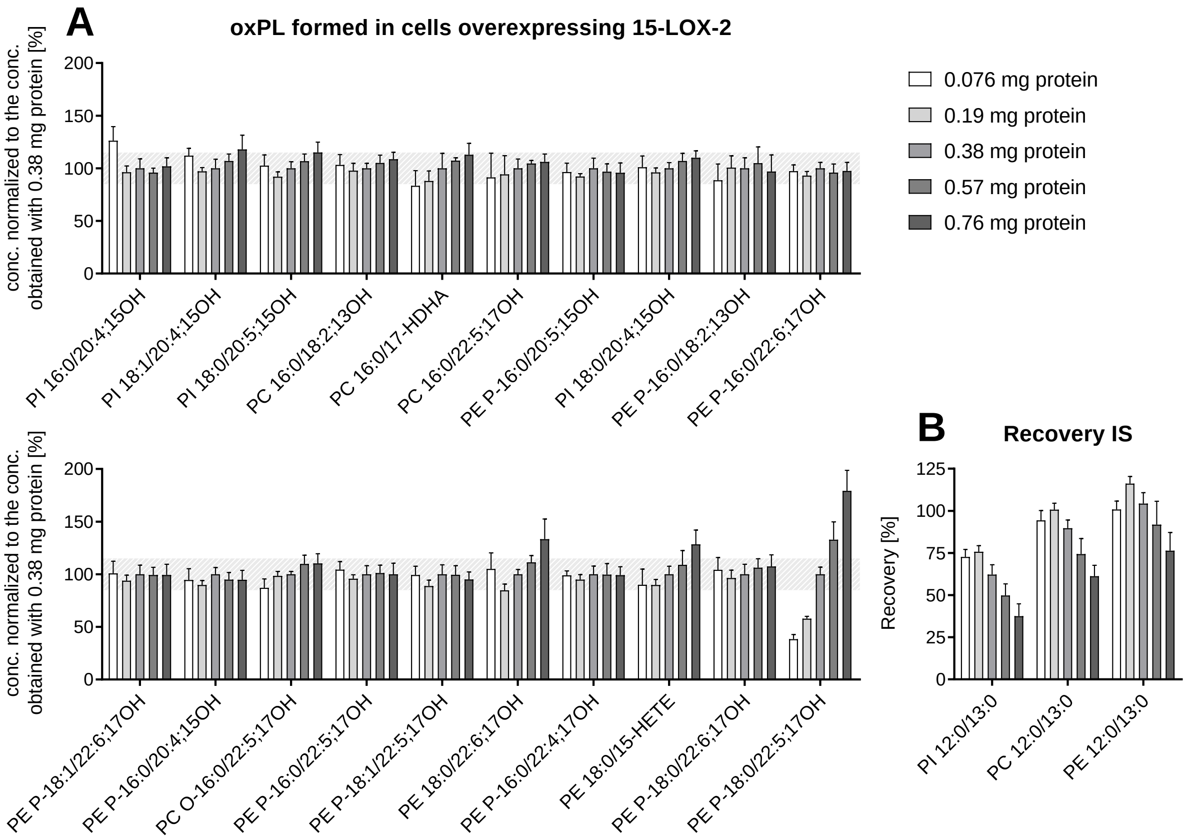

Fig. S3: Effect of the number of cells (mg cellular protein) on the apparent concentration and the recovery of ISs. Endogenous formation of oxPLs elicited by overexpression of 15-LOX-2 in 5 × 10^6 HEK293T_15-LOX-2 cells. Extraction was performed using different volumes of cell homogenate — 10 µL (0.076 mg protein), 25 µL (0.19 mg protein), 50 µL (0.38 mg protein), 75 µL (0.57 mg protein), and 100 µL (0.76 mg protein) — while maintaining identical extraction solvent volumes. Analytes are sorted by retention time. Shown is the mean ± SD (n = 3) of (A**)** the concentrations normalized to the one obtained with 0.38 mg protein and (B**)** the recovery of the ISs. The concentration of each oxPL can be found in Table S5.

Overall, varying protein amounts used for extraction yielded consistent concentrations for almost all oxPLs, with a few exceptions, emphasizing the robustness of the developed LC-MS/MS method. Good recoveries of ISs and no apparent matrix effects were observed within the elution window of 7 to 18 min when 0.38 mg protein or less was used for LLE. Extraction using 0.38 mg protein also enabled the detection of more oxPL species compared to lower protein amounts. Therefore, a sample amount of 0.38 mg cellular protein was selected for extraction, as it minimized matrix interferences while enabling the sensitive detection of oxPLs.

Extraction efficiency

OxPLs are present in much lower concentrations compared to unoxidized PLs in biological samples, and thus their detection can be challenging. Selection of a suitable extraction procedure from biological samples is a key prerequisite to ensure good extraction recovery and coverage of oxPLs with varying polarity.

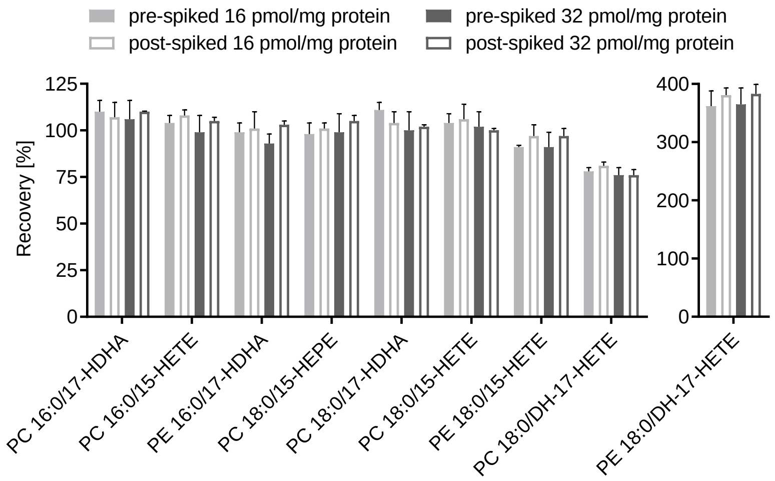

Fig. S4: Extraction efficiency of oxPL spiked in HEK293T cells. Shown are the recoveries of spiked oxPL from HEK293T cell homogenates. A mixture of oxPL was added to the cell sample (6.25 or 12.5 pmol of each) at the beginning of sample preparation (pre-spiked) or after sample preparation directly before LC-MS/MS analysis (post-spiked). OxPL are sorted by retention time. Shown are mean values ± SD, (n = 3).

MTBE-based LLE effectively extracts both unoxidized PL (Fig. S2) and oxPL species (Fig. S4) differing in polar head groups and fatty acyl chain composition. Comparison of recoveries of oxPL spiked to cell samples prior to or post-extraction showed losses of less than 10% during extraction for all oxPC and oxPE tested (Fig. S4). The addition of oxPLs before LC-MS/MS analysis unveiled ion suppression/enhancement effects for the two last eluting PLs bearing dihomo-17-hydroxyeicosatetraenoic acid (DH-17-HETE): while only 20% of the signal of PC 18:0/DH-17-HETE was suppressed, PE 18:0/DH-17-HETE was subjected to a strong enhancement effect of almost 400%, probably caused by coelution with abundant PE P-18:0/22:5;17OH (Fig. S3A). Thus, these two lipid species can be analyzed by the LC-MS/MS method after dilution (see below). Other analytes eluting from 11 to 17 min were not affected by matrix effects. Intra- and inter-day variability were excellent with <100 ± 14% for almost all analytes and slightly lower for PC 18:0/17-HDHA with ≤ 100 ± 19% (Table S6).

All in all, these results indicate that MTBE-based LLE is well suited for the extraction of oxPL species in HEK293T cells. Previous studies extracted oxPLs using a solvent mixture of acetic acid/IPA/*n*-hexane (3, 6) or using solid-phase extraction with C18 cartridges (4, 5), but did not present data about extraction recovery. In this study, the sample preparation used for oxPLs is simple, environmentally friendly, and efficiently extracts lipids without discrimination of lipid species, enabling the investigation of both PLs and oxPLs by a single extraction procedure.

Intra- and inter-day accuracy and precision

Intra- and inter-day accuracy and precision of the analytical procedure were characterized based on the Guideline on bioanalytical method validation of the International Council for Harmonization (8). In order to evaluate the accuracy and precision, HEK293T cell samples spiked at four concentration levels were analyzed in three replicates on three different days (Table S7).

Excellent intra- and inter-day accuracy and precision were obtained for analytes eluting from 11 min to 17 min, while later-eluting analytes i.e., PC/PE bearing 18:0 and DH-17-HETE, were detected with lower accuracy (Table S7). Indeed, except for PC/PE bearing DH-17-HETE, the intra-day accuracy ranged from 75% to 112% indicating low interference of cell matrix in the elution window from 11 to 17 min. For PC 18:0/DH-17-HETE (t_R = 18.3 min), inter- and intra-day accuracy was lower but still acceptable, ranging from 66% to 80%, indicating moderate ion suppression effects. For all analytes, intra-day precision calculated as RSD was <15% at all spiked levels with two exceptions (18% and 21%) over 108 data points calculated. Inter-day precision was better, below 9% indicating a stable method enabling the analysis of large sample batches.

Our results show that most oxPLs can be analyzed with excellent accuracy. Only the signal of oxPLs eluting after 17 min could be disturbed by severe matrix effects, as shown for PE 18:0/DH-17-HETE. Lipidomics analysis typically relies on a single IS to cover multiple analytes (3-7), which does not correct for possible matrix effects in reversed-phase chromatography. Also, in our approach only few, non-oxidized PLs are used as ISs (see above). Thus, matrix effects cannot be fully compensated by the IS, which emphasizes the importance of assessing oxPL accuracy as shown here.

Dilution integrity of the determined concentration for oxidized glycero-PL

Extraction recoveries and accuracy evaluations showed a strong enhancement effect on PE 18:0/DH-17-HETE (Fig. S3A, Fig. S4), while other analytes exhibited excellent accuracy and recoveries. To further characterize and support the absence of matrix effects, we evaluated the accuracy and extraction recoveries of oxPLs and ISs added at the beginning of the sample preparation in non-diluted and diluted lipid extracts (Fig. S5).

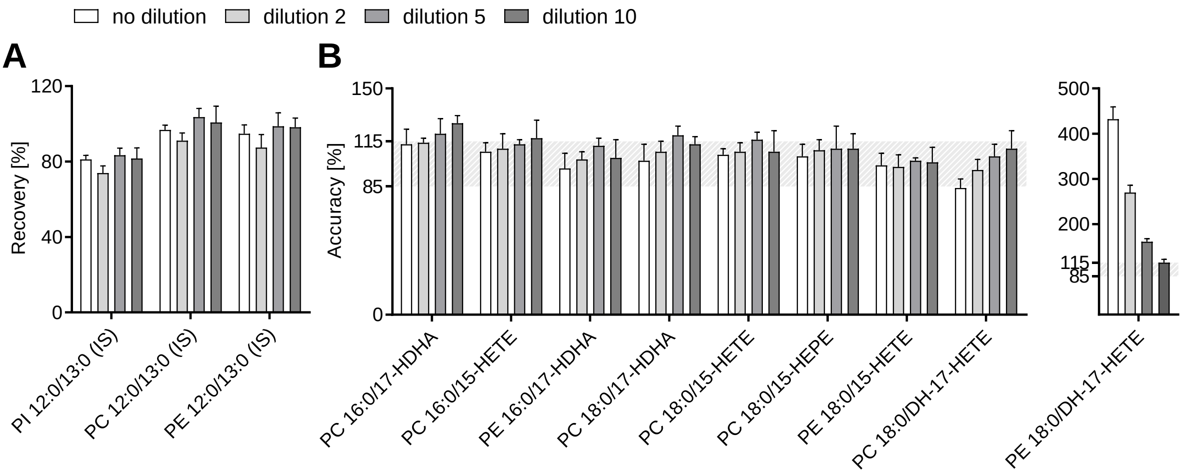

Fig. S5: Dilution integrity of IS and spiked oxPL in HEK293T cells. Cell homogenates (0.38 mg protein) were spiked with a mixture of oxPL (12.5 pmol of each) and IS (2.5 pmol of each) prior to extraction. Lipid extracts were diluted by a factor of 2, 5, or 10 prior to targeted LC-MS/MS analysis. Shown are in non-diluted and diluted cell extracts (A) the recovery of IS and (B) the accuracy of the spiked oxPL determined from the calculated concentration (mean ± SD (n = 3)). Analytes are sorted by retention time.

No ion suppression effects are present for analytes eluting from 7.4 to 17 min (Fig. S5). However, ion suppression or ion enhancement effects were observed for analytes eluting after 18 min (Fig. S5B). Calculated extraction recoveries of IS were nearly identical in non-diluted and diluted lipid extracts (Fig. S5A). Also, remarkable constant concentrations were observed across different dilutions of the lipid extract, with an accuracy ranging from 97% and 120% (Fig. S5B), except for PC 16:0/17-HDHA with an accuracy of 127% in the 1:10 diluted extract close to the LLOQ. This supports the data on the accuracy and precision (Table S7). Increasing dilution improved the accuracy of the two late-eluting PLs bearing DH-17-HETE (Fig. S5B), indicating a reduction of ion matrix effects: For PC 18:0/DH-17-HETE, the accuracy increased from 84% in non-diluted extract to 110% in the 1:10 diluted extract. Similarly, the accuracy of PE 18:0/DH-17-HETE was dramatically improved by dilution: Starting from 432%, to 270%, 161%, and finally 115% in the 1:10 diluted extract. Thus, a 1:10 dilution also enabled accurate quantification for these two-lipid species.

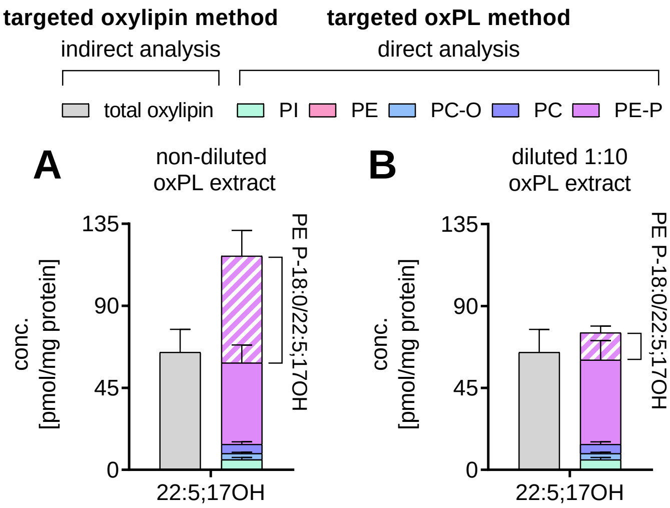

Fig. S6: Dilution of oxPL extract improves quantification accuracy by reducing ion suppression of PE P-18:0/22:5;17OH. Esterified oxylipins were quantified in 15-LOX-2 overexpressing HEK293T_15-LOX-2 cells (0.38 mg protein) either following alkaline hydrolysis or as intact oxPL by targeted LC-MS/MS methods. Shown is the comparison of the concentration of 22:5;17OH in (A) non-diluted oxPL extract and (B) 1:10 diluted oxPL extract. (Grey bar) Total 22:5;17OH quantified following alkaline hydrolysis. (Colored bar) sum of individual oxPL species of each lipid class. Shown is the mean ± SD per mg cellular protein (n = 3)**.**

The 1:10 dilution of the oxPL extract (Fig. S6) reduced the matrix effects on PE 18:0/22:5;17OH formed via 15-LOX-2 in HEK293T_15-LOX-2 cells. The calculated concentration of PE P-18:0/22:5;17OH dropped from 61 pmol/mg protein in non-diluted extract (Fig. S6A) to 15 pmol/mg in the 1:10 diluted extract (Fig. S6B). After dilution, the resulting concentration of (the sum of) PLs bearing 22:5;17OH was nearly identical compared to the targeted oxylipin method, with only a 15% difference (Fig. S6B). This demonstrates the accuracy of the quantification of PE P-18:0/22:5;17OH by targeted LC-MS/MS using an extraction volume of 50 µL (0.38 mg protein) and a 1:10 dilution.

All in all, these results support the occurrence of ion suppression/enhancement effects after 18 min but also demonstrate that a factor 10 dilution of the oxPL extract can resolve these matrix effects, consistent with the literature (9). Additionally, the consistent concentrations observed for all other analytes across different dilutions of the oxPL extract underline the robustness of the developed method and the absence of matrix interferences.

Overexpression of 15-LOX-2 in HEK293T_15-LOX-2 cells

The successful induction of 15-LOX-2 expression in HEK293T_15-LOX-2 cells was confirmed by combined targeted metabolomics/proteomics (1) (Fig. S7).

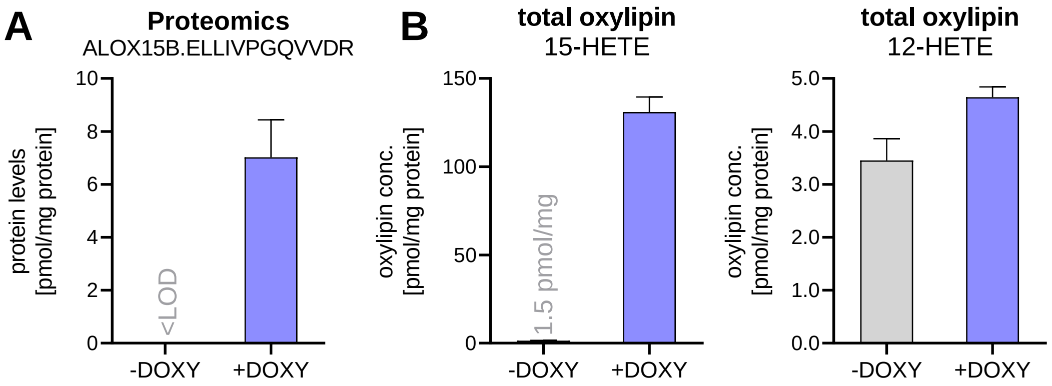

Fig. S7: Characterization of 15-LOX-2 overexpression induced by doxycycline in HEK293T cells transfected with 15-LOX-2 using combined targeted proteomics and oxylipin metabolomics. Shown are (A) protein level of expressed 15-LOX-2 and (B) total oxylipin concentration of (left) 15-HETE and (right) 12-HETE of the HEK293T cells inducible transfected with 15-LOX-2 with and without incubation of (24 h, 200 ng/mL) doxycycline (DOXY). *ALOX15B* peptides were measured following tryptic digestion by targeted LC-MS/MS proteomics. Total oxylipins were analyzed following alkaline hydrolysis by targeted LC-MS/MS. Shown is the mean concentration ± SD per mg cellular protein (n = 3)**.**

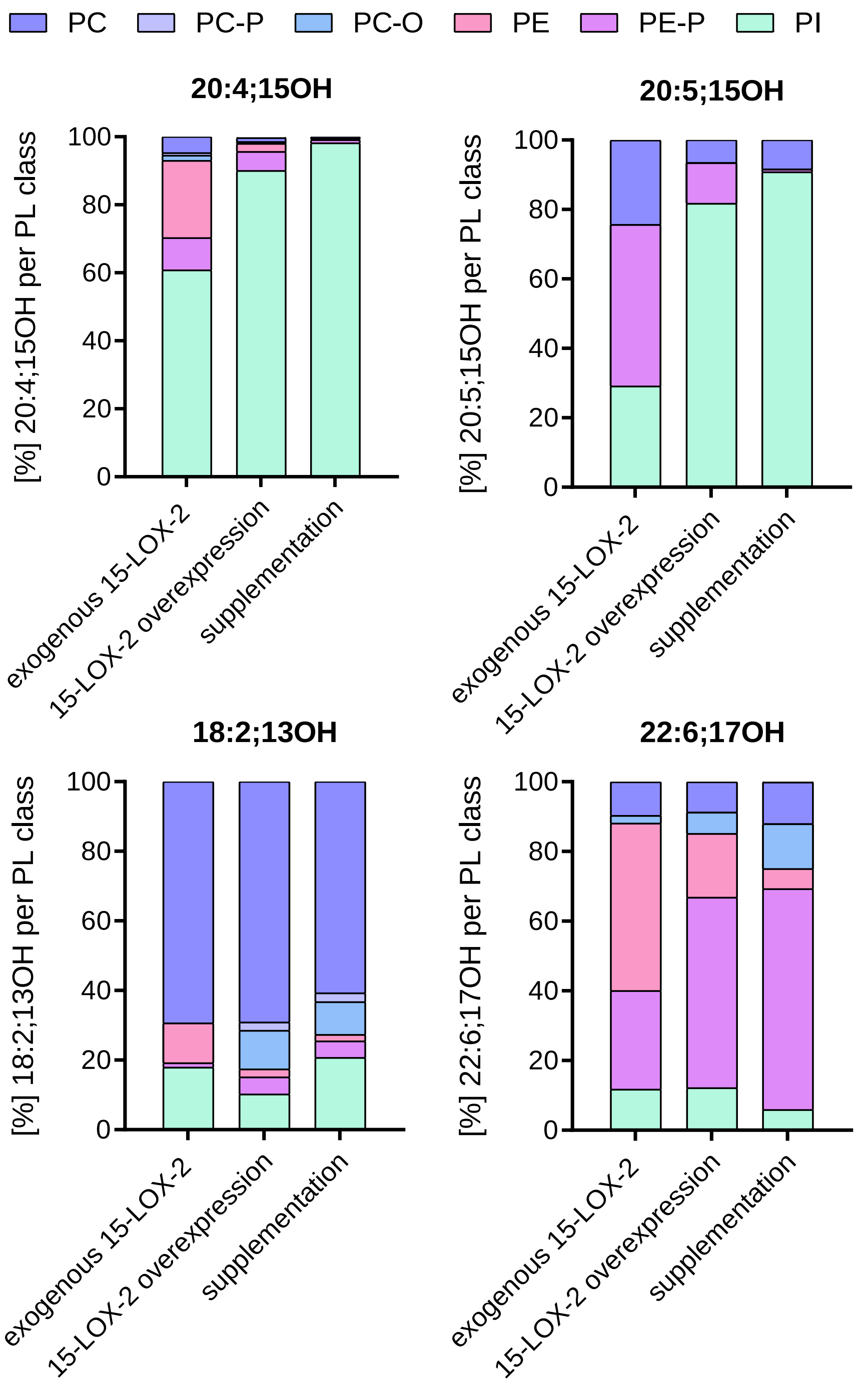

Fig. S8: Investigation of the oxPL pattern formed by exogenous 15-LOX-2 added to the cell homogenate versus endogenous formation by 15-LOX-2 or incorporation following supplementation. Shown is the relative distribution of 20:4;15OH, 20:5;15OH, 22:6;17OH, and 18:2;13OH per PL class in HEK293T cells. **(Left bar)** 15-LOX-2 human enzyme (6.9 × 10^{-3} U/mL in assay) was added to cell homogenate of HEK293T cells and incubated for 2 h at 37 °C. **(Middle bar)** Endogenous formation of oxPLs was elicited by overexpression of 15-LOX-2 in HEK293T_15-LOX-2 (200 ng/mL doxycycline, 24 h). **(Right bar)** HEK293T cells were supplemented with either 15(*S*)-HETE, 15(*S*)-HEPE, 17(*S*)-HDHA, or 13(*S*)-HODE (300 nM, 2 h). For each oxylipin, data were normalized on the sum of all oxPLs quantified by targeted LC-MS/MS. Shown is the mean ± SD (n = 3).

References

1. Hartung NM, Mainka M, Pfaff R, Kuhn M, Biernacki S, Zinnert L, et al. (2023) Development of a quantitative proteomics approach for cyclooxygenases and lipoxygenases in parallel to quantitative oxylipin analysis allowing the comprehensive investigation of the arachidonic acid cascade. *Anal Bioanal Chem*. **415**, 913-33.
2. Hartung NM, Mainka M, Kampschulte N, Ostermann AI, Schebb NH (2019) A strategy for validating concentrations of oxylipin standards for external calibration. *Prostaglandins & Other Lipid Mediators*. **141**, 22-4.
3. Slatter DA, Aldrovandi M, O'Connor A, Allen SM, Brasher CJ, Murphy RC, et al. (2016) Mapping the human platelet lipidome reveals cytosolic phospholipase A2 as a regulator of mitochondrial bioenergetics during activation. *Cell metabolism*. **23**, 930-44.
4. Aoyagi R, Ikeda K, Isobe Y, Arita M (2017) Comprehensive analyses of oxidized phospholipids using a measured MS/MS spectra library. *J Lipid Res*. **58**, 2229-37.
5. Nakanishi H, Iida Y, Shimizu T, Taguchi R (2009) Analysis of oxidized phosphatidylcholines as markers for oxidative stress, using multiple reaction monitoring with theoretically expanded data sets with reversed-phase liquid chromatography/tandem mass spectrometry. *J Chromatography B*. **877**, 1366-74.
6. Morgan AH, Dioszeghy V, Maskrey BH, Thomas CP, Clark SR, Mathie SA, et al. (2009) Phosphatidylethanolamine-esterified eicosanoids in the mouse: tissue localization and inflammation-dependent formation in Th-2 disease. *J Biolog Chem*. **284**, 21185-91.
7. Hammad LA, Wu G, Saleh MM, Klouckova I, Dobrolecki LE, Hickey RJ, et al. (2009) Elevated levels of hydroxylated phosphocholine lipids in the blood serum of breast cancer patients. *Rapid Communications in Mass Spectrometry: An International Journal Devoted to the Rapid Dissemination of Up-to-the-Minute Research in Mass Spectrometry*. **23**, 863-76.
8. Ojha A, Bhargava S. (2022) International council for harmonisation (ICH) guidelines. Regulatory affairs in the pharmaceutical industry: Elsevier. p. 47-74.
9. Stahnke H, Reemtsma T, Alder L (2009) Compensation of matrix effects by postcolumn infusion of a monitor substance in multiresidue analysis with LC− MS/MS. *Analytical chemistry*. **81**, 2185-92.
